# Astrocytic response mediated by the CLU risk allele inhibits OPC proliferation and myelination in a human iPSC model

**DOI:** 10.1016/j.celrep.2023.112841

**Published:** 2023-07-25

**Authors:** Zhenqing Liu, Jianfei Chao, Cheng Wang, Guihua Sun, Daniel Roeth, Wei Liu, Xianwei Chen, Li Li, E Tian, Lizhao Feng, Hayk Davtyan, Mathew Blurton-Jones, Markus Kalkum, Yanhong Shi

**Affiliations:** 1 Department of Neurodegenerative Diseases, Beckman Research Institute of City of Hope, Duarte, CA 91010, USA; 2 Department of Molecular Imaging and Therapy, Beckman Research Institute of City of Hope, Duarte, CA 91010, USA; 3 Department of Immunology, Hebei Medical University, Shijiazhuang, Hebei 050017, China; 4 Department of Neurobiology & Behavior, Institute for Memory Impairments & Neurological Disorders and Sue & Bill Gross Stem Cell Research Center, University of California Irvine, Irvine, CA 92697, USA; 5 Lead contact

## Abstract

The C allele of rs11136000 variant in the clusterin (*CLU*) gene represents the third strongest known genetic risk factor for late-onset Alzheimer’s disease. However, whether this single-nucleotide polymorphism (SNP) is functional and what the underlying mechanisms are remain unclear. In this study, the CLU rs11136000 SNP is identified as a functional variant by a small-scale CRISPR-Cas9 screen. Astrocytes derived from isogenic induced pluripotent stem cells (iPSCs) carrying the “C” or “T201D allele of the CLU rs11136000 SNP exhibit different CLU expression levels. TAR DNA-binding protein-43 (TDP-43) preferentially binds to the “C” allele to promote CLU expression and exacerbate inflammation. The interferon response and CXCL10 expression are elevated in cytokine-treated C/C astrocytes, leading to inhibition of oligodendrocyte progenitor cell (OPC) proliferation and myelination. Accordingly, elevated CLU and CXCL10 but reduced myelin basic protein (MBP) expression are detected in human brains of C/C carriers. Our study uncovers a mechanism underlying reduced white matter integrity observed in the CLU rs11136000 risk “C” allele carriers.

## In brief

Liu et al. show that the CLU rs11136000 is a functional variant by a small-scale CRISPR-Cas9 screen. Astrocytes derived from iPSCs carrying the risk allele “C” exhibit elevated CLU expression and inflammatory response. Cytokine-treated C/C astrocytes inhibit OPC proliferation and myelination through activating the interferon response and inducing CXCL10 expression.

## Graphical Abstract

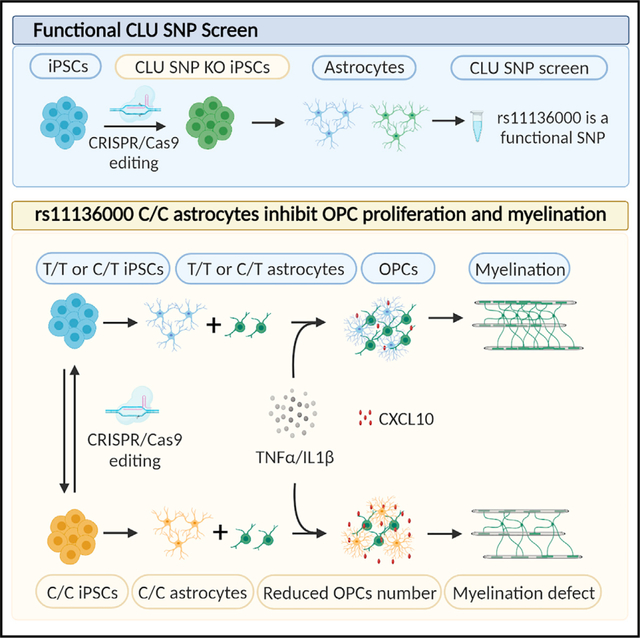

## INTRODUCTION

Alzheimer’s disease (AD) is the most common form of dementia in the elderly with no cure.^1,2^ Mechanisms underlying AD pathogenesis remain to be elucidated to develop effective therapies for this disease. AD research using animal models has greatly facilitated our understanding of this disease. Because of significant species differences in brain physiology and lifespan between human and rodents, establishing human models will complement animal studies to better understand AD. The human induced pluripotent stem cell (hiPSC) platform has provided an opportunity to generate human brain cells for studying brain development and disease. Since the advent of the induced pluripotent stem cell (iPSC) technology,^3,4^ hiPSCs have been rapidly applied to model diseases,^3–6^ including AD.^7–11^

Most patients with AD have sporadic late-onset AD.^12^ The C allele of the rs11136000 single-nucleotide polymorphism (SNP) in the clusterin (*CLU*) gene represents the third strongest known genetic risk factor for late-onset AD,^13,14^ with the more prevalent C allele conferring greater AD risk, while the less common T allele considered protective.^13,14^ Like apolipoprotein E (ApoE), *CLU* encodes a major brain apolipoprotein.^15^ CLU has been shown to exhibit functions in lipid transport^16^ and metabolism,^17,18^ Aβ deposition and clearance,^19–22^ amyloid plaque formation and neuritic dystrophy,^20,23–25^ tau pathology,^24–26^ oxidative stress and neurotoxicity,^27^ synaptic degeneration,^28^ and neuroinflammatory response.^20,29–32^ Although there is a strong association of the C allele of the rs11136000 SNP with increased risk for AD, mechanisms underlying the risk effect of this *CLU* SNP remain to be determined.

CLU is expressed in astrocytes and neurons, with higher levels in astrocytes.^33–35^ Growing evidence supports the idea that astrocytes play an important role in regulating myelination.^36–39^ The interplay between astrocytes and oligodendrocyte progenitor cells (OPCs) could modulate oligodendrocyte homeostasis and myelination. Increasing studies have implicated white matter abnormalities in AD.^40^ Changes at radiological, pathological, and molecular levels have been observed in the white matter of patients with AD,^40,41^ which are thought to reflect demyelination and axon damage.^42^ Moreover, single-cell transcriptomic analysis of brains from patients with AD has highlighted myelination-related processes in AD pathogenesis.^43^ Myelination-related genes have been shown to be perturbed in the brain of patients with AD.^43^ Although myelin abnormalities can be associated with cognitive deterioration in AD, mechanisms underlying myelin changes in AD remain to be explored.

In this study, we identified the widely studied CLU SNP rs11136000 as a functional variant by CRISPR-Cas9 knockout (KO) of this SNP and adjacent SNPs that exhibit high linkage equilibrium with it. Moreover, by switching the risk “C” or the protective “T” allele using CRISPR-Cas9 editing, we generated isogenic iPSCs that have different alleles of the CLU rs11136000 SNP. Astrocytes derived from the isogenic iPSCs carrying the “C” or “T” allele exhibited different CLU expression and differential inflammatory response following cytokine treatment. We determined how astrocytes carrying the “C” or “T” allele regulate OPC proliferation and myelination in response to cytokine treatment and identified a molecular mechanism underlying this regulation.

## RESULTS

### Identifying the rs11136000 SNP of the *CLU* gene as a functional variant

**Figure 1.**
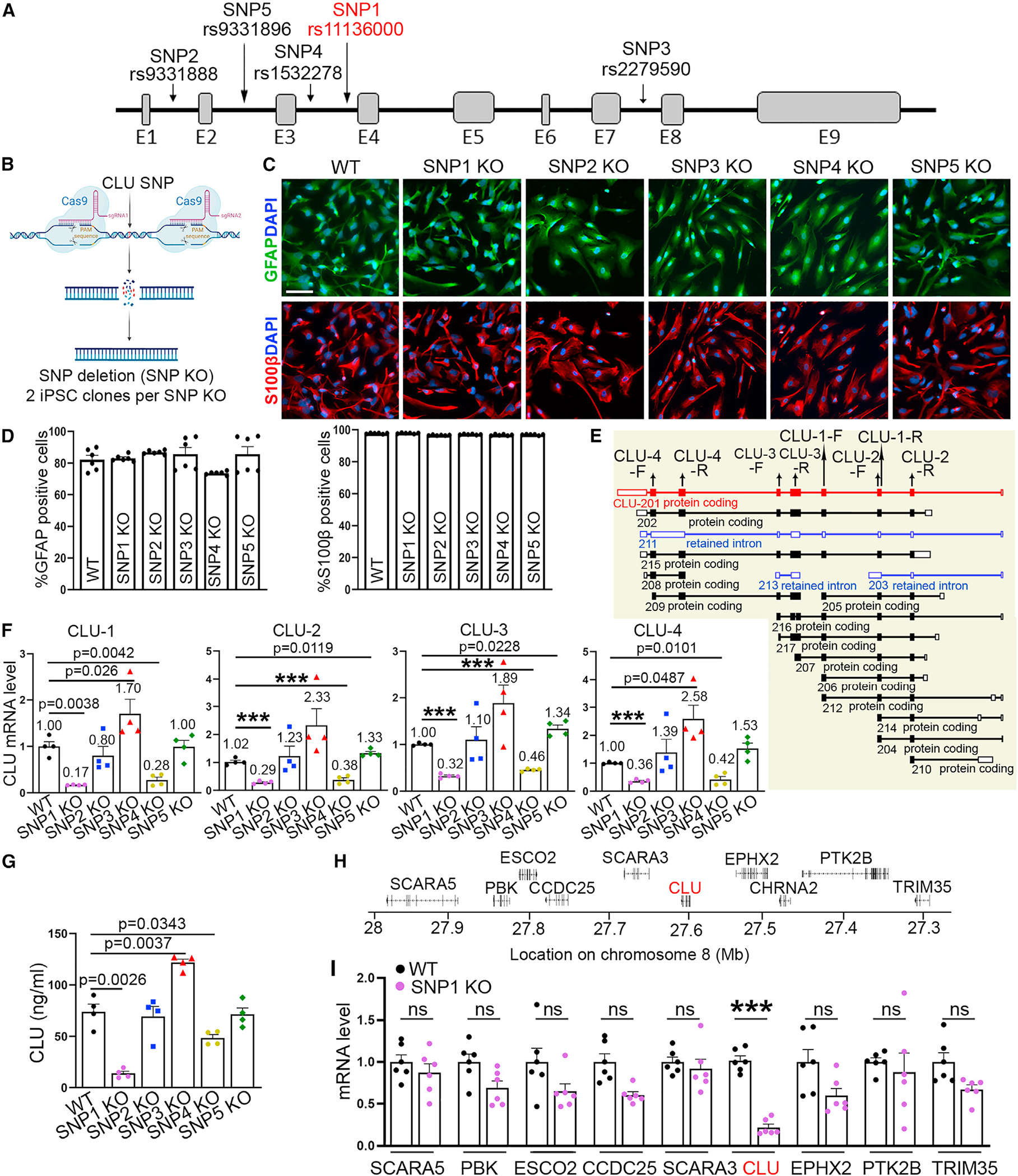
Deletion of the CLU rs11136000 SNP region reduces CLU expression level in astrocytes (A) A schematic depicting the location of 5 CLU SNPs. (B) A schematic showing CRISPR-Cas9-based CLU SNP KO. (C and D) The purity of the WT or SNP KO astrocytes shown by GFAP or S100β staining (C). The quantification is shown in (D). (E) A schematic showing 17 CLU isoforms and the location of 4 pairs of CLU primers. (F) Reduced CLU mRNA level in SNP1 KO astrocytes revealed by qRT-PCR. (G) Reduced secreted CLU protein level from SNP1 KO astrocytes revealed by ELISA. (H) A schematic depicting the genomic region containing *CLU* and neighboring genes. (I) Reduced mRNA level of CLU but not surrounding genes in SNP1 KO astrocytes revealed by qRT-PCR. Error bars are SEM of the mean. p values are indicated or labeled ***(p < 0.001), analyzed using one-way ANOVA followed by unpaired t test with Welch’s correction for (F) and (G) and two-way ANOVA followed by Bonferroni’s multiple-comparison test for (I). ns, not statistically significant (p > 0.05). n = 6 images per group for (D), 4 independent experiments for (F) and (G), and 6 independent experiments for (I). For (F) and (I), the mRNA level relative to that in WT astrocytes is shown. Scale bar: 50 μm for (C).

Although the rs11136000 variant (SNP1) is the most widely studied CLU SNP, it is not clear whether it is a functional variant, because there are several CLU SNPs, including rs9331888 (SNP2), rs2279590 (SNP3), rs1532278 (SNP4), and rs9331896 (SNP5) (Figure 1A), that exhibit high linkage disequilibrium with the rs11136000 SNP.^13,14^ Multiple studies have shown elevated CLU expression in brains from patients with AD or brain regions affected by AD.^19,25,26,44–49^ To identify functional variant(s) among the rs11136000 SNP and its surrounding variants that exhibit high linkage disequilibrium, we examined these variants for their role in regulating CLU expression by a small-scale CRISPR-Cas9 screen. We performed CRISPR-Cas9 editing in hiPSCs to knock out regions spanning these CLU variants (SNP1-5) individually (Figure 1B). All iPSC lines with the SNP region KO (termed SNP KO in short) exhibited normal karyotype (Tables S1 and S2). No off-target effect from CRISPR-Cas9 editing was detected in SNP KO iPSCs (Table S3). The gene-edited clones were confirmed by genomic DNA sequencing (Figure S1A). Because CLU is highly expressed in astrocytes,^35^ the resultant SNP KO iPSCs were differentiated into astrocytes by transducing iPSC-derived neural progenitor cells (NPCs) with lentivirus encoding the astrocyte transcription factors SRY-box transcription factor 9 (SOX9), nuclear factor I A (NFIA), and nuclear factor I B (NFIB).

The resultant astrocytes expressed astrocyte markers glial fibrillary acidic protein (GFAP), S100 calcium binding protein B (S100β), and SOX9, with about 80% GFAP^+^ cells, nearly 100% S100β^+^ cells, and more than 80% SOX9^+^ cells (Figures 1C, 1D, S1B, and S1E). Four pairs of *CLU* primers were designed to detect the expression of 16 overlapping *CLU* isoforms out of total 17 isoforms (Figure 1E). Compared with wild-type (WT) astrocytes, we detected significantly reduced CLU expression level in SNP1 KO and SNP4 KO astrocytes with all 4 pairs of *CLU* primers, elevated CLU expression level in SNP3 KO and SNP5 KO astrocytes with 1 or 2 pairs of *CLU* primers, and no significant change in *CLU* expression in SNP2 KO astrocytes with all 4 pairs of *CLU* primers (Figure 1F). The CLU expression level change exhibited the same trend by qRT-PCR using four distinct primer pairs in SNP1 KO and SNP4 KO astrocytes (Figures 1E and 1F), suggesting that the CLU mRNA level change in these SNP KO astrocytes is likely due to an overall decrease in transcription. Accordingly, ELISA analysis revealed decreased level of secreted CLU protein in SNP1 KO and SNP4 KO astrocytes, but increased level in SNP3 KO astrocytes (Figure 1G). The decreased CLU expression in SNP1 KO and SNP4 KO astrocytes and increased CLU expression in SNP3 KO astrocytes suggest that the SNP1 and SNP4 regions may contain stimulatory elements while the SNP3 region may harbor an inhibitory element for CLU expression. Because the SNP2 KO line went through the same editing process but exhibited no statistically significant change in CLU expression level (Figures 1F and 1G), the phenotype of CLU expression level change in SNP1, SNP3, and SNP4 KO astrocytes was likely not due to the editing process but resulted from KO of the SNP region. These results suggest that the *CLU* SNP1, SNP3, and SNP4 are likely functional variants. Because the highest fold change of CLU expression level was seen in SNP1 KO astrocytes (Figures 1F and 1G), we focused the following studies on the *CLU* SNP1 (rs11136000). Examining the expression level of genes surrounding the *CLU* locus on the same chromosome revealed that the SNP1 KO specifically reduced the expression of *CLU* but not surrounding genes (Figures 1H and 1I), suggesting a specific role for SNP1 in regulating CLU expression.

### The C allele of the *CLU* rs11136000 SNP is associated with elevated CLU expression

**Figure 2.**
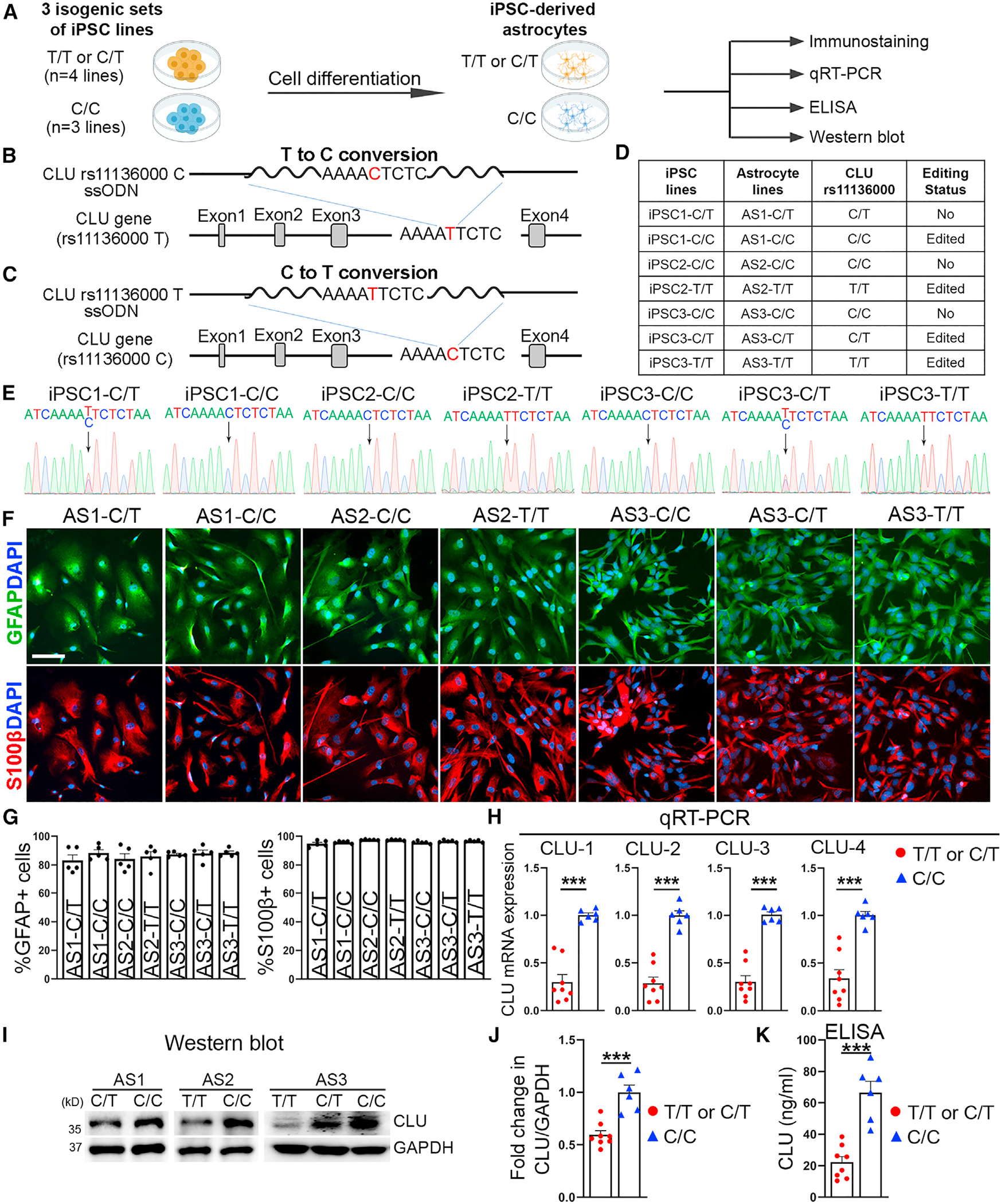
CLU rs11136000 C/C astrocytes exhibit higher CLU expression level (A) A schematic summarizing the experimental design. (B and C) A schematic showing CRISPR-Cas9 editing to convert “T” to “C” (B) or “C” to “T” (C) in the CLU rs11136000 SNP. (D) A table listing parental or gene-edited C/C, C/T, or T/T iPSCs and astrocytes. (E) Sanger sequencing of CLU rs11136000 in parental or gene-edited iPSCs. (F and G) The purity of the CLU rs11136000 SNP-carrying astrocytes shown by GFAP or S100β staining (F). The quantification is shown in (G). (H–J) Elevated CLU expression in C/C astrocytes compared with T/T or C/T astrocytes as revealed by qRT-PCR (H) or western blot (I and J). The CLU mRNA or protein level in T/T or C/T astrocytes relative to their isogenic C/C astrocytes is shown. (K) Elevated level of secreted CLU protein from C/C astrocytes compared with T/T or C/T astrocytes as revealed by ELISA. Error bars are SEM of the mean. ***p < 0.001, analyzed using two-tailed Student’s t test for (H), (J), and (K). n = 5 images per line for (G), n = 8 for T/T or C/T (4 iPSC lines, 2 batches of differentiation per line), and 6 for C/C (3 iPSC lines, 2 batches of differentiation per line) for (H), (J), and (K). Scale bar: 50 μm for (F).

It has been shown that the C allele of the *CLU* rs11136000 variant confers greater AD risk, whereas the T allele is protective.^13,14^ To define the effect of the C and the T allele unequivocally, we performed CRISPR-Cas9 editing to convert “C” to “T” or “T” to “C” in hiPSCs (Figures 2A–2C; Table S1). To avoid complications from different ApoE genotypes, we used hiPSC lines from ApoE3/3 carriers only. We obtained three sets of isogenic iPSC lines, including 3 C/C lines and 4 C/T or T/T lines (Figure 2D). The genotype of the edited clones was confirmed by genomic DNA sequencing (Figure 2E). All isogenic iPSC lines exhibited normal karyotype (Table S1; Figure S1C). No off-target effect from CRISPR-Cas9 editing was detected (Table S3).

We then differentiated these isogenic iPSCs into astrocytes. The resultant astrocytes expressed GFAP, S100β and SOX9, with more than 80% GFAP^+^ cells, nearly 100% S100β^+^ cells, and more than 80% SOX9^+^ cells (Figures 2F, 2G, S1D, and S1F). C/C astrocytes exhibited similar proliferative rate to T/T or C/T astrocytes (Figure S1G). To confirm whether the *CLU* rs11136000 SNP regulates CLU expression, we performed RNA sequencing (RNA-seq) using RNAs isolated from two pairs of isogenic astrocytes (AS1 [C/C vs. C/T] and AS2 [C/C vs. T/T]). Elevated CLU expression was detected in C/C astrocytes, compared with T/T or C/T astrocytes on RNA-seq. The differential CLU expression in astrocytes carrying only the risk “C” allele (C/C) vs. astrocytes carrying the protective “T” allele (C/T or T/T) was confirmed using three sets of isogenic astrocytes, including 3 lines of C/C astrocytes and 4 lines of C/T or T/T astrocytes listed in Figure 2D. Increased CLU expression in C/C astrocytes, compared with T/T or C/T astrocytes, was confirmed by qRT-PCR (Figure 2H) and western blot analyses (Figures 2I and 2J). The specificity of the CLU antibody was revealed by western blot using WT and CLU KO iPSC-derived brain organoids (Figure S1H). ELISA analysis of astrocyte conditioned medium revealed elevated level of secreted CLU in C/C astrocytes, compared with T/T or C/T astrocytes (Figure 2K). These results together indicate that the *CLU* rs11136000 SNP plays a role in regulating CLU expression, with the C allele associated with elevated CLU expression.

**Figure 3.**
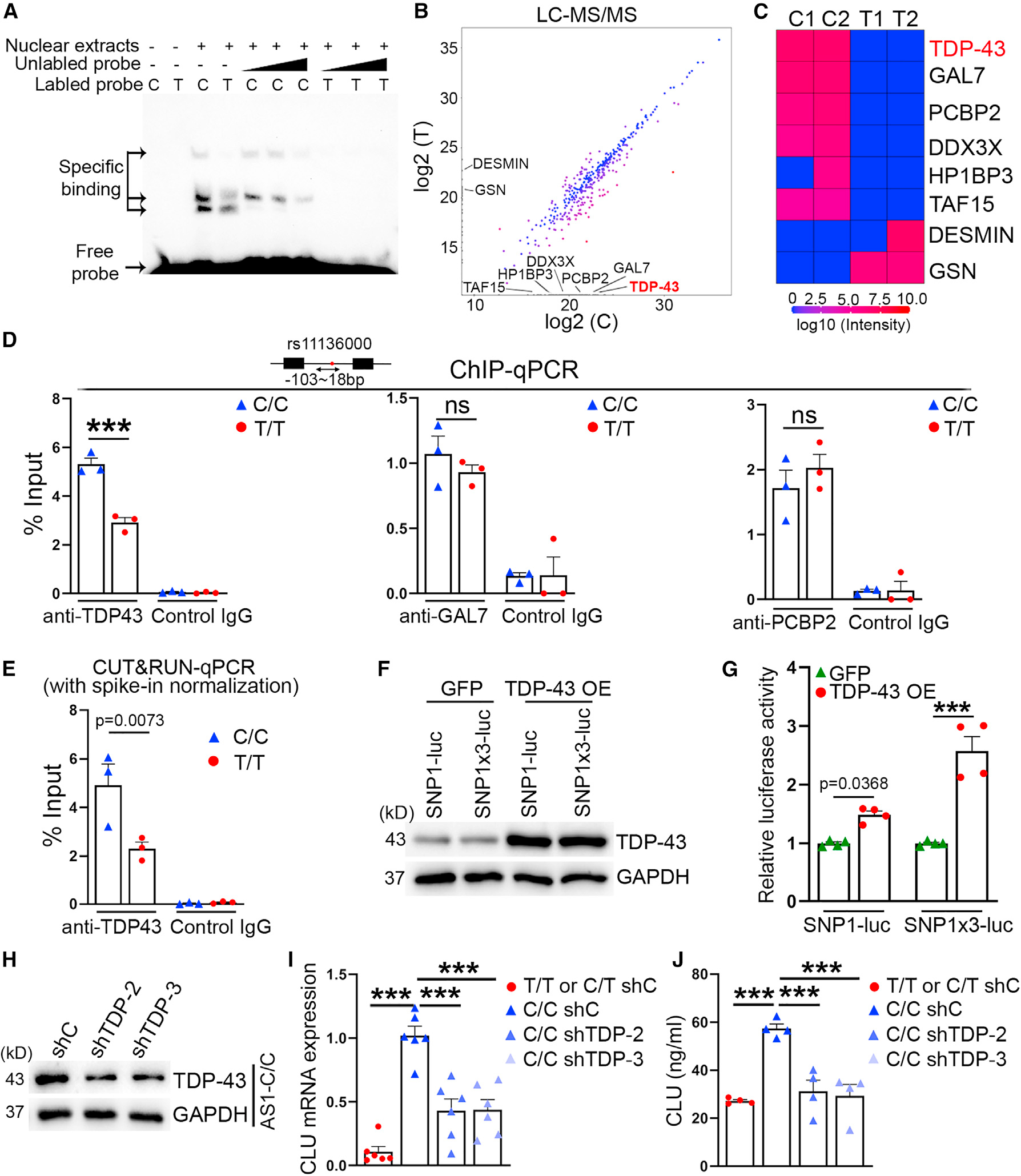
Differential binding of TDP-43 to the CLU rs11136000 SNP with the “C” or “T” allele (A) Gel shift assay showing binding of astrocyte nuclear extracts to CLU rs111366000 C/C or T/T DNA sequences. (B and C) Scatterplot (B) and heatmap (C) showing representative proteins that exhibited differential binding to C/C or T/T SNP. (D) ChIP-qPCR assay showing differential binding of TDP-43 to the CLU rs11136000 SNP in C/C and T/T astrocytes. The binding of GAL7 and PCBP2 was analyzed in parallel. (E) Differential binding of TDP-43 to CLU rs11136000 in C/C or T/T astrocytes was validated using CUT&RUN-qPCR with spike-in normalization. (F) Western blot showing overexpression (OE) of TDP-43 protein in HEK293T cells transfected with CLU SNP1-luc or SNP1×3-luc reporter together with TDP-43. (G) Luciferase reporter assay showing induction of firefly luciferase reporter downstream of CLU SNP1 by TDP-43 OE. (H) Western blot showing knockdown of TDP-43 protein in TDP-43 shRNA (shTDP-2 or shTDP-3)-transduced astrocytes. shC, control shRNA. (I) qRT-PCR showing reduced CLU mRNA and secreted protein level in C/C astrocytes transduced with TDP-43 shRNA. The CLU mRNA level relative to that in C/C astrocytes transduced with shC is shown. (J) ELISA showing reduced level of secreted CLU protein in C/C astrocytes transduced with TDP-43 shRNA. Error bars are SEM of the mean. p values are indicated or labeled ***(p < 0.001), analyzed using two-way ANOVA followed by Bonferroni’s multiple-comparison test for (D), (E), and (G) and one-way ANOVA followed by Tukey’s multiple-comparison test for (I) and (J). ns, not statistically significant (p > 0.05). n = 3 independent experiments for each condition for (D) and (E), 4 independent experiments for each condition in (G) and (J), and 6 independent experiments for each condition in (I).

How does the *CLU* rs11136000 SNP regulate CLU expression? We hypothesized that regulatory factors may bind to the *CLU* SNP with the “C” or “T” allele differentially, which leads to differential CLU expression. To test this hypothesis, we performed electrophoretic mobility shift assay, also called gel shift assay, that has been used to study protein-DNA interactions, by incubating biotin-labeled *CLU* rs11136000 SNP DNA sequence containing either the “C” or “T” allele with nuclear extracts of human astrocytes. We detected protein complexes associated with the biotin-labeled *CLU* SNP containing the “C” or “T” allele (Figure 3A, lanes 3 and 4). Addition of excess unlabeled *CLU* SNP oligoes containing either the “C” or “T” allele was able to compete away the biotin-labeled *CLU* SNP in the complexes in a dose-dependent manner (Figure 3A, lanes 5–10), indicating that the binding is specific. Of note, we were able to detect different intensity of nuclear extracts binding to the “C” allele vs. the “T” allele-containing SNP (Figure 3A, lanes 3 and 4), indicating that nuclear proteins can bind to the *CLU* SNP with the “C” or “T” allele differentially.

To identify nuclear factors that exhibit differential binding to the *CLU* SNP with the “C” or “T” allele, we conjugated the biotin-labeled *CLU* SNP with the “C” or “T” allele to streptavidin magnetic beads and incubated nuclear extracts of human primary astrocytes with the beads. After extensive washes, bound proteins were eluted and subjected to liquid chromatography-tandem mass spectrometry (LC-MS/MS) analysis. Of 538 protein groups detected in LC-MS/MS, 158 proteins were predicted to be nuclear localized. A list of nuclear proteins exhibited differential binding to the “C” vs. the “T” allele as shown in the scatter-plot and heatmap (Figures 3B and 3C; Table S4). To validate differential binding to the “C” vs. the “T” allele by the top candidates from LC-MS/MS, we performed chromatin immunoprecipitation (ChIP)-qPCR using C/C or T/T astrocytes. We detected substantially more TAR DNA-binding protein-43 (TDP-43) binding to the *CLU* rs11136000 SNP in C/C astrocytes compared with that in T/T astrocytes, whereas no statistically significant difference was detected in binding of galactose-1-phosphate uridylyltransferase (GAL7) and poly(RC) binding protein 2 (PCBP2) to the *CLU* rs11136000 SNP in C/C and T/T astrocytes (Figure 3D). The differential binding of TDP-43 to the *CLU* rs11136000 SNP in C/C vs. T/T astrocytes was confirmed by CUT&RUN-qPCR (Figure 3E). These results indicate that TDP-43 can bind to the “C” or “T” allele in the *CLU* rs11136000 SNP differentially.

To determine if binding of TDP-43 to the *CLU* SNP1 modulates CLU expression, we prepared a reporter construct in which a firefly luciferase reporter gene was placed downstream of one copy of *CLU* SNP1 (SNP1-luc) or 3 copies of *CLU* SNP1 (SNP1×3-luc). We transfected a TDP-43 or a GFP-expressing vector together with SNP1-luc or SNP1×3-luc into HEK293T cells. The transfection efficiency of GFP plus SNP1-luc and GFP plus SNP1×3-luc was 98.2% and 98.5%, respectively (Figure S2B). Overexpression of TDP-43 led to increased luciferase reporter activity and the increase was more dramatic with SNP1×3-luc than that with SNP1-luc (Figures 3F and 3G), indicating that TDP-43 can promote CLU expression through the CLU SNP1.

To determine if TDP-43 regulates CLU expression in astrocytes, we knocked down TDP-43 in astrocytes by small hairpin interfering RNAs (shRNA). C/C astrocytes were transduced with lentivirus encoding the TDP-43 shRNA or a control shRNA. The lentiviral transduction efficiency was evaluated by quantifying the percentage of the GFP reporter-positive cells and more than 80% transduction efficiency was detected for each condition (Figure S2C). Knockdown (KD) of TDP-43 was confirmed by western blot (Figure 3H). Decreased TDP-43 expression led to reduced CLU mRNA and secreted protein level in C/C astrocytes (Figures 3I and 3J). These results together indicate that TDP-43 can bind to the *CLU* rs11136000 SNP with the “C” or “T” allele differentially to regulate CLU expression in astrocytes.

### C/C astrocytes exhibit exacerbated interferon response

**Figure 4.**
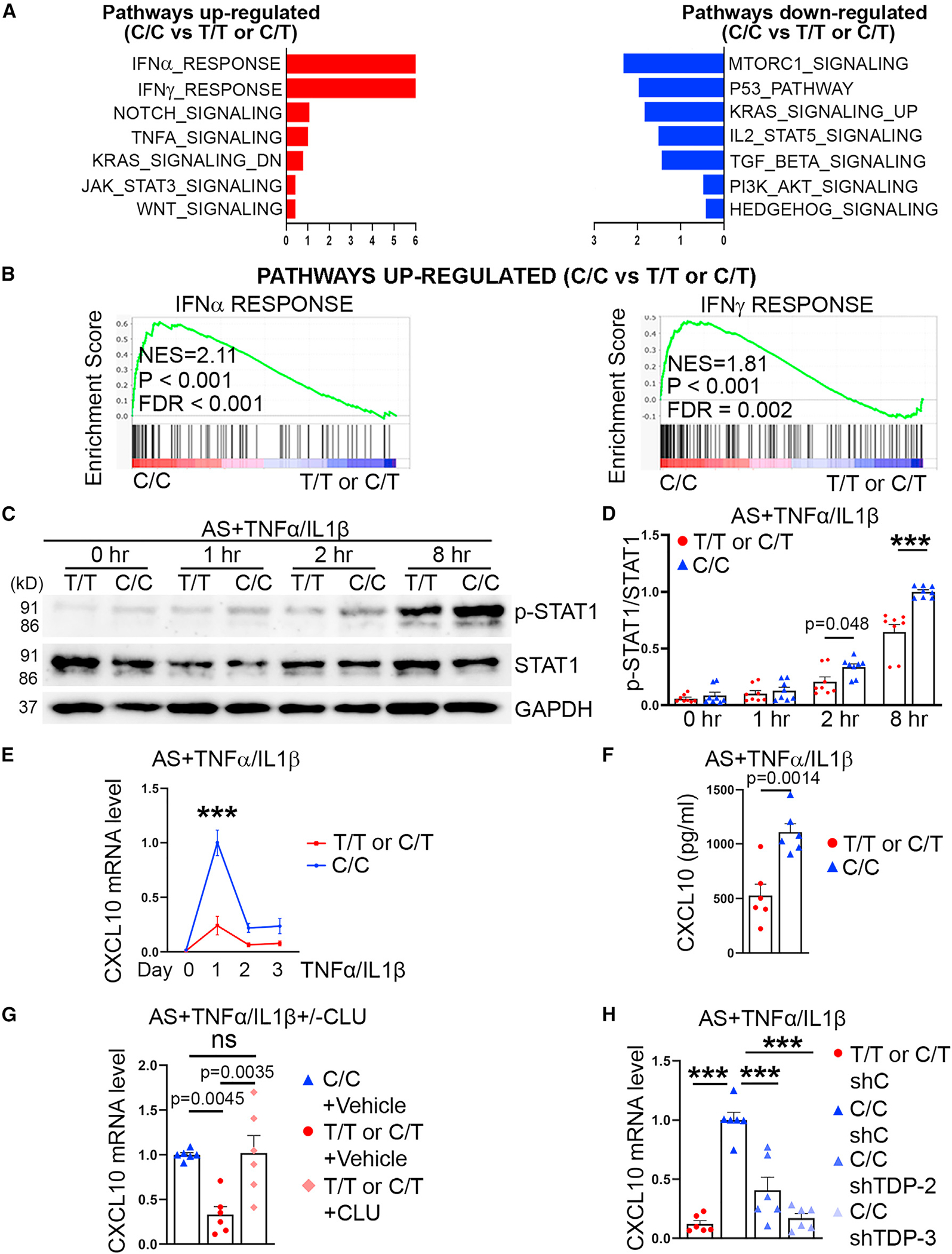
C/C astrocytes exhibit enhanced IFN response and elevated CXCL10 expression in response to cytokine stimulation (A and B) C/C astrocytes exhibit enhanced IFN response. Up-regulated or down-regulated pathways in C/C astrocytes, compared with T/T or C/T astrocytes, are shown in (A). Positive correlation of C/C astrocytes with IFNα and IFNγ response is shown in (B). (C and D) Elevated level of p-STAT1 in TNF-α/IL-1β-treated C/C astrocytes revealed by western blot. T/T or C/T and C/C astrocytes were treated with TNF-α/IL-1β for 0, 1, 2, and 8 h. The relative ratio of p-STAT1 to total STAT1 is shown in (D). (E) Elevated induction of CXCL10 mRNA level in TNF-α/IL-1β-treated C/C astrocytes revealed by qRT-PCR. The CXCL10 mRNA level was normalized to that in C/C astrocytes treated with TNF-α/IL-1β for 1 day. (F) ELISA detection of CXCL10 protein levels in astrocytes treated with TNF-α/IL-1β for 1 day. (G) qRT-PCR showing elevated CXCL10 mRNA levels in T/T or C/T astrocytes treated with CLU. Data were normalized to that in vehicle-treated C/C astrocytes(with TNF-α/IL-1β treatment). (H) qRT-PCR showing reduced CXCL10 mRNA levels in TNF-α/IL-1β-treated C/C astrocytes transduced with TDP-43 shRNA. Data were normalized to that in TNF-α/IL-1β-treated C/C astrocytes transduced with control shRNA (shC). Error bars are SEM of the mean. p values are indicated or labeled ***(p < 0.001), analyzed using two-way ANOVA followed by Bonferroni’s multiple-comparison test for (D) and (E), two-tailed Student’s t test for (F), and one-way ANOVA followed by Tukey’s multiple-comparison test for (G) and (H). ns, not statistically significant (p > 0.05). Two isogenic pairs were used, with 2 iPSC lines per group. Data relative to isogenic C/C astrocytes are shown in (D), (E), (G), and (H). n = 8 independent experiments for (D) and (E) and 6 independent experiments for (F)–(H).

To identify genes that were differentially expressed in astrocytes carrying only the risk “C” allele (C/C) vs. astrocytes carrying the protective “T” allele (C/T or T/T), we subjected two pairs of isogenic astrocytes (AS1 [C/C vs. C/T] and AS2 [C/C vs. T/T]) to RNA-seq, in which AS1-C/C and AS2-C/C contain only the risk “C” allele, while AS1-C/T and AS2-T/T are carriers of the protective “T” allele. The up-regulated genes were defined as genes that have the fold change ≥ 2 and p < 0.05 in C/C vs. T/T or C/T astrocytes, while the down-regulated genes were defined as genes that have the fold change ≥ 0.5 and p < 0.05. On the basis of these criteria, 271 genes were up-regulated and 176 genes were down-regulated in C/C astrocytes. Pathway analysis revealed the interferon (IFN) response pathway as the top pathway up-regulated in C/C astrocytes, compared with T/T or C/T astrocytes (Figures 4A and 4B). Because IFN signals through activation of the JAK-STAT pathway,^50^ we determined the activation status of STAT1 in C/C vs. T/T or C/T astrocytes treated for a time course of 8 h with tumor necrosis factor alpha (TNF-α)/interleukin-1 beta (IL-1β), mimicking the inflammatory milieu with elevated levels of TNF-α/IL-1β in brains from patients with AD.^51,52^ Activation of STAT1, as revealed by increased level of phosphorylated STAT1 (p-STAT1), was detected in both C/C and T/T or C/T astrocytes upon TNF-α/IL-1β stimulation with stronger activation in C/C astrocytes (Figures 4C and 4D), indicating that C/C astrocytes exhibit heightened IFN response following cytokine treatment.

C-X-C motif chemokine ligand 10 (CXCL10) is an IFNγ-inducible chemokine^53^ that plays an important role in neuroinflammation.^54,55^ The elevated induction of IFN response by cytokine stimulation in C/C astrocytes led us to determine if expression of IFN-inducible genes is differentially regulated in C/C vs. C/T or T/T astrocytes upon cytokine treatment. We determined the expression of a set of cytokines/chemokines that are expressed by astrocytes, including IFN-inducible cytokines/chemokines CXCL10, CXCL1, and CCL5,^56^ in TNF-α/IL-1β-treated C/C vs. T/T or C/T astrocytes (Figures 4E and S2). Among the cytokines/chemokines examined, the induction of CXCL10 exhibited the highest induction in C/C astrocytes compared with T/T or C/T astrocytes one day after TNF-α/IL-1β treatment (Figures 4E and S2). The level of CXCL10 protein was also higher in conditioned medium of C/C astrocytes than that in T/T or C/T astrocytes treated with TNF-α/IL-1β (Figure 4F). These results indicate that C/C astrocytes express and release a higher level of CXCL10 than T/T or C/T astrocytes when treated with cytokine.

To determine if increased CLU level is causal to elevated CXCL10 induction by cytokines in C/C astrocytes compared with T/T or C/T astrocytes, we treated T/T or C/T astrocytes with human CLU protein. Treatment with CLU led to elevated CXCL10 induction in TNF-α/IL-1β-treated T/T or C/T astrocytes, to a level similar to that in TNF-α/IL-1β-treated C/C astrocytes (Figure 4G). On the other hand, KD of TDP-43, which reduced CLU expression (Figure 3J), resulted in decreased CXCL10 expression level in TNF-α/IL-1β-treated C/C astrocytes (Figure 4H). These results indicate that C/C astrocytes exhibit heightened IFN response, which leads to elevated CXCL10 induction by cytokines.

### C/C astrocytes inhibit OPC proliferation

**Figure 5.**
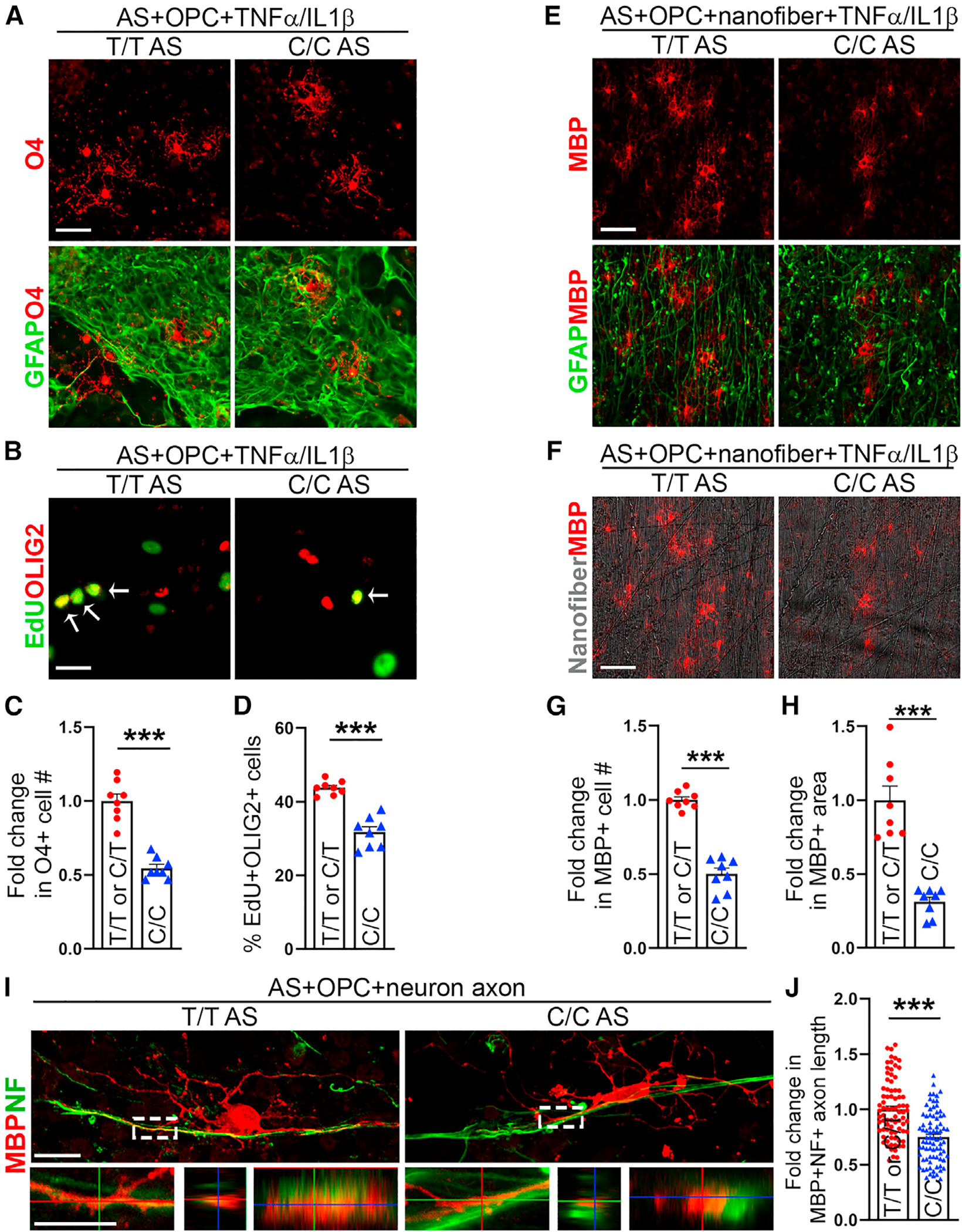
Cytokine-treated C/C astrocytes inhibit OPC proliferation and myelination (A and C) Cytokine-treated C/C astrocytes reduced O4^+^ OPC number 6 days after co-culture. The fold change (C) is relative to O4^+^ cell number in co-cultures with T/T or C/T astrocytes. (B and D) Cytokine-treated C/C astrocytes inhibit OPC proliferation. The proliferation of OPC was evaluated by EdU labeling 1 day after co-culture. Arrows point to the EdU^+^OLIG2^+^ cells. Quantification is shown in (D). (E–H) Cytokine-treated C/C astrocytes reduce the number of MBP^+^ oligodendrocytes. Astrocyte-OPC-nanofiber co-cultures were stained for MBP 20 days after co-culture. The fold change (G and H) is relative to data in co-cultures with T/T or C/T astrocytes. (I and J) C/C astrocytes in astrocyte-neuron-OPC co-cultures reduce MBP^+^NF^+^ axon length. The axon length was evaluated 20 days after co-culture. Higher magnification images are shown in the lower panels. The fold change (J) is relative to data in co-cultures with T/T or C/T astrocytes. Error bars are SEM of the mean. ***p < 0.001, analyzed using two-tailed Student’s t test for (C), (D), (G), (H), and (J). Two isogenic pairs were used, with 2 iPSC lines per group. n = 8 independent experiments for (C), (D), (G), and (H); n = 80 MBP^+^ cells from 6 independent experiments for (J). Scale bar: 50 μm for (A), (B), (E), and (F), 20 μm for (I) (upper panel), and 10 μm for (I) (lower panel).

IFN response has been shown to drive neuroinflammation in AD^57^ and neuroinflammation is associated with myelin damage.^58–60^ CXCL10 has been shown to inhibit myelination.^61–63^ Moreover, results from human brain imaging study have shown reduced myelination in C/C brains compared with T/T or C/T brains.^64^ Therefore, we hypothesized that C/C astrocytes could cause myelination defects through release of elevated CXCL10 upon cytokine treatment. To test this hypothesis, we first evaluated the effects of CXCL10 protein on OPC proliferation. hiPSCs from a healthy subject (I90) were differentiated into protein O4^+^ OPCs and purified using O4-based magnetic-activated cell sorting (MACS).^65,66^ These OPCs were treated with different doses of CXCL10. Reduced OPC proliferation was detected in cells treated with 100 or 1,000 pg/mL CXCL10 (Figure S4A). Then we established astrocyte-OPC co-cultures using C/C or T/T (C/T) astrocytes together with I90 OPCs with the CLU SNP1 C/T genotype and subjected the co-cultures to TNF-α/IL-1β treatment. A decrease in the number of O4^+^ OPCs was detected after 6 day co-culture with C/C astrocytes, compared with co-culture with T/T or C/T astrocytes (Figures 5A and 5C). These results indicate that cytokine-treated C/C astrocytes could reduce OPC number in astrocyte-OPC co-cultures.

To test whether the decrease in the number of OPCs was due to a decrease in OPC proliferation or an increase in cell apoptosis, we determined the rate of OPC proliferation and apoptosis in co-cultures containing C/C vs. T/T or C/T astrocytes that were treated with TNF-α/IL-1β. For OPC proliferation, cells were treated with 5-ethynyl-2′-deoxyuridine (EdU) on day 2 of co-culture. A decrease in the percentage of EdU^+^ oligodendrocyte transcription factor 2 (OLIG2)^+^ and EdU^+^ SRY-box transcription factor 10 (SOX10)^+^ OPCs was detected in co-cultures with C/C astrocytes, compared with that in co-cultures with T/T or C/T astrocytes (Figures 5B, 5D, S4B, and S4C). A mild reduction in OPC proliferation and O4^+^ OPC number was also detected in astrocyte-OPC co-cultures with C/C vs. T/T or C/T astrocytes without TNF-α/IL-1β treatment, but the reduction was more modest compared with that observed after TNF-α/IL-1β treatment (Figures S3A–S3D).

To detect OPC apoptosis, the astrocyte-OPC co-cultures were subjected to TNF-α/IL-1β treatment for 1 day. Then cells were double stained for cleaved caspase-3 (Cas3) and SOX10. No significant difference in the percentage of Cas3^−^SOX10^+^ OPCs was detected in co-cultures with C/C astrocytes, compared with that in co-cultures with T/T or C/T astrocytes (Figures S4D and S4E). These results indicate that cytokine-treated C/C astrocytes can inhibit OPC proliferation.

### C/C astrocytes reduce MBP^+^ oligodendrocyte number and MBP^+^ NF^+^ axon length

Next, we asked if cytokine-treated C/C astrocytes could reduce myelinating oligodendrocyte number, leading to myelination defects. To address this question, we co-cultured OPCs with C/C vs. T/T or C/T astrocytes on nanofibers in OPC medium and then switched to oligodendrocyte maturation medium.^66–68^ We observed decreased number of myelin basic protein (MBP)^+^ oligodendrocytes and reduced area of MBP-covered nanofibers in co-cultures with C/C astrocytes under cytokine treatment, compared with that in co-cultures with T/T or C/T astrocytes under the same condition (Figures 5E–5H). A moderate reduction in the number of MBP^+^ oligodendrocytes and area of MBP-covered nanofibers was also detected in co-cultures with C/C astrocytes compared with that in co-cultures with T/T or C/T astrocytes without TNF-α/IL-1β treatment (Figures S3E–S3G), but the reduction was much milder than that observed with TNF-α/IL-1β treatment (Figures 5E–5H). To test whether the decrease in the number of MBP^+^ oligodendrocytes was due to an increase in oligodendrocyte apoptosis, we double stained co-cultured cells with Cas3 and MBP 20 days after co-culture. No significant difference in the percentage of Cas3^−^MBP^+^ oligodendrocytes was detected in co-cultures with C/C astrocytes compared with that in co-cultures with T/T or C/T astrocytes under cytokine treatment (Figures S4F and S4G).

When we seeded astrocyte-OPC co-cultures directly in oligodendrocyte maturation medium, we were still able to detect reduced MBP^+^ cell number and MBP^+^ area in co-cultures with C/C astrocytes compared with that in co-cultures with T/T or C/T astrocytes (Figure S4H), although the extent of decrease was less than what we detected when we seeded co-cultures in OPC medium and then switched to maturation medium (Figures 5G and 5H). These results suggest that C/C astrocytes can reduce both OPC proliferation and maturation.

To further determine the effect of C/C astrocytes on myelination, we established astrocyte-neuron-OPC co-cultures. Elevated CXCL10 protein level was detected in astrocyte-neuron-OPC co-cultures with C/C astrocytes compared with that in co-cultures with T/T or C/T astrocytes (Figure S4I). The differential CXCL10 expression in astrocyte-neuron-OPC co-cultures with C/C vs. T/T or C/T astrocytes without cytokine treatment could be resulted from different extent of enrichment in matrisome genes through distinct astrocyte-neuron communications.^69^ Myelination in co-cultures was evaluated by measuring the MBP^+^ neurofilament H (NF)^+^ axon length. We detected reduced MBP^+^NF^+^ axon length in co-cultures with C/C astrocytes compared with that in co-cultures with T/T or C/T astrocytes (Figures 5I and 5J), supporting the idea that C/C astrocytes can inhibit myelination.

### KD of TDP-43 in C/C astrocytes leads to increased OPC proliferation and myelination in astrocyte-OPC co-cultures

To determine the functional relevance of TDP-43-mediated regulation, we transduced C/C astrocytes with lentivirus encoding the TDP-43 shRNA (TDP-43 KD astrocytes) or a control shRNA (control astrocytes). The transduced astrocytes were co-cultured with OPCs and the co-cultures were treated with TNF-α/IL-1β. TDP-43 KD in C/C astrocytes increased OPC proliferation and O4^+^ cell number (Figures S5A–S5C). Accordingly, increased MBP^+^ cell number and MBP^+^ area were detected in astrocyte-OPC co-cultures on nanofibers with TDP-43 KD C/C astrocytes compared with that in co-cultures with control C/C astrocytes treated with TNF-α/IL-1β (Figures S5D–S5E). Moreover, increased MBP^+^NF^+^ axon length was detected in astrocyte-neuron-OPC co-cultures with TDP-43 KD C/C astrocytes compared with that in co-cultures with control C/C astrocytes (Figures S5F and S5G). These results indicate that astrocytic TDP-43 is an important regulator of OPC proliferation, myelinating oligodendrocyte number and myelination.

### A CXCL10-neutralizing antibody rescues OPC proliferation and myelination defects in astrocyte-OPC co-cultures

To determine if TNF-α/IL-1β-treated C/C astrocytes inhibit OPC proliferation via secreted molecules, we collected conditioned medium from TNF-α/IL-1β-treated C/C and T/T (or C/T) astrocytes and applied the conditioned medium to OPCs. We included conditioned medium from astrocytes without TNF-α/IL-1β treatment as a control. Conditioned medium from C/C astrocytes without TNF-α/IL-1β treatment induced no obvious difference in OPC proliferation and O4^+^ cell number compared with conditioned medium from T/T or C/T astrocytes without TNF-α/IL-1β treatment (Figures S6A–S6C). In contrast, conditioned medium from TNF-α/IL-1β-treated C/C astrocytes inhibited OPC proliferation and reduced O4^+^ OPC number, compared with conditioned medium from TNF-α/IL-1β-treated T/T or C/T astrocytes (Figures S6D–S6F). These results indicate that molecules secreted from TNF-α/IL-1β-treated C/C astrocytes can inhibit OPC proliferation and reduce OPC number.

**Figure 6.**
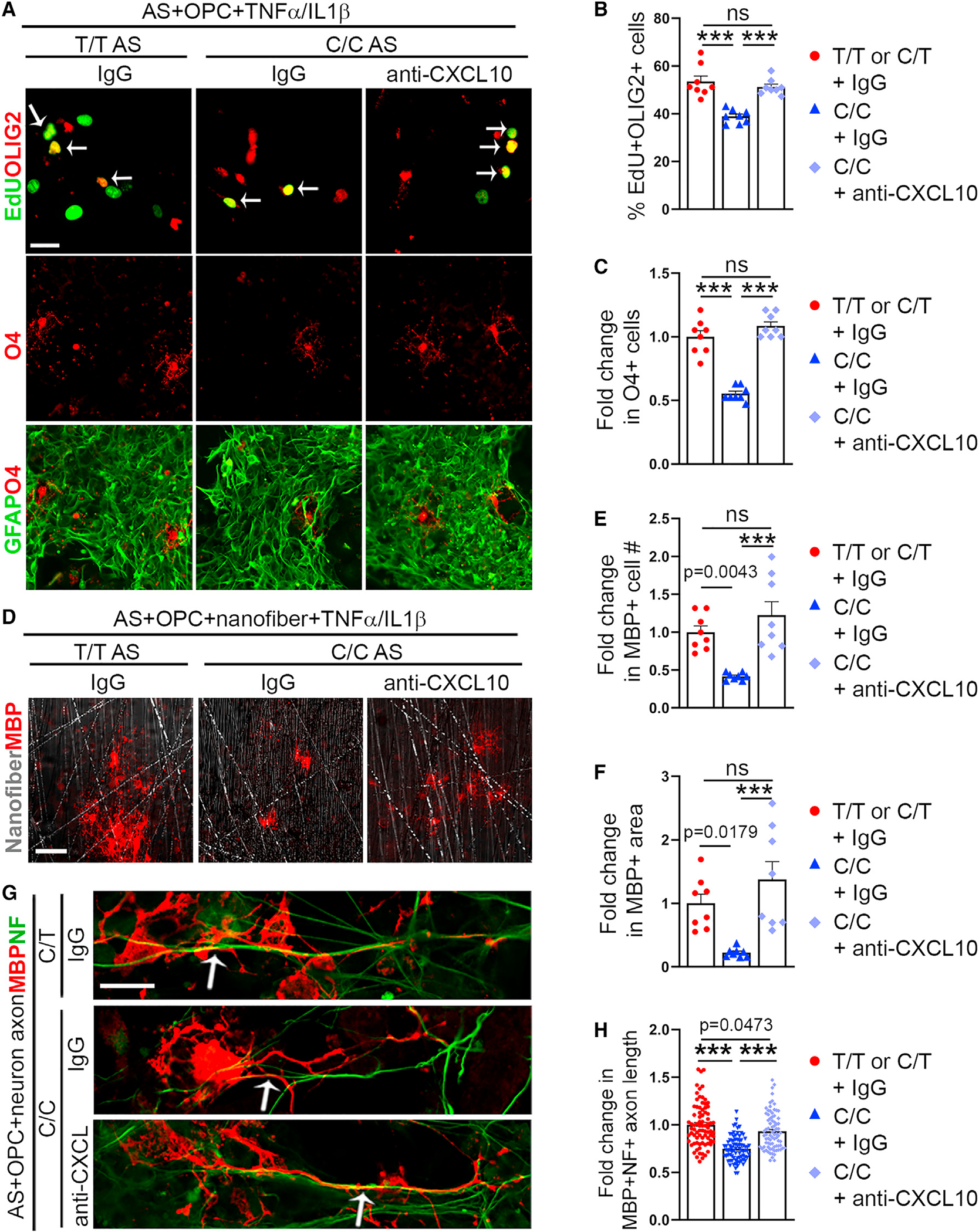
CXCL10 mediates the inhibitory effect of C/C astrocytes on OPC proliferation and myelination (A–C) Treatment with the CXCL10-neutralizing antibody rescues OPC proliferation in astrocyte-OPC co-cultures with C/C astrocytes treated with TNF-α/IL-1β. Representative images of EdU and OLIG2 double staining after 1 day co-culture (upper panels), O4 staining or O4 and GFAP double staining after 6 day co-culture (lower panels) in astrocyte-OPC co-cultures with indicated treatment. The percentage of EdU^+^OLIG2^+^ cells is shown in (B) and the fold change in O4^+^ cell number in C. The fold change is relative to data in co-cultures with T/T or C/T astrocytes treated with IgG plus TNF-α/IL-1β. (D–F) Treatment with the CXCL10-neutralizing antibody rescues MBP^+^ cell number and area in astrocyte-OPC co-cultures with C/C astrocytes treated with TNF-α/IL-1β. Representative images of MBP staining and nanofiber in astrocyte-OPC-nanofiber co-cultures 20 days after co-culture. The fold change in MBP^+^ cell number (E) and area (F) is relative to data in T/T or C/T astrocyte-OPC-nanofiber co-cultures treated with IgG plus TNF-α/IL-1β. (G and H) Treatment with the CXCL10-neutralizing antibody rescues MBP^+^NF^+^ axon length in astrocyte-neuron-OPC co-cultures with C/C astrocytes. Representative images of MBP and NF co-staining of astrocyte-neuron-OPC co-cultures 20 days after co-culture (G). Examples of the MBP^+^NF^+^ axons are pointed by arrows. The fold change in MBP^+^NF^+^ axon length (H) is relative to data in co-cultures with T/T or C/T astrocytes treated with IgG. Error bars are SEM of the mean. p values are indicated or labeled ***(p < 0.001), analyzed using one-way ANOVA followed by Tukey’s multiple-comparison test. ns, not statistically significant (p > 0.05). Two isogenic pairs were used, with 2 iPSC lines per group. n = 8 independent experiments for (B), (C), (E), (F), n = 80 MBP^+^ cells from 6 independent experiments for (H). Scale bar: 50 μm for (A) and (D) and 20 μm for (G).

To determine if elevated CXCL10 induction by TNF-α/IL-1β in C/C astrocytes is essential for reduced OPC proliferation in astrocyte-OPC co-cultures with C/C astrocytes, we treated C/C astrocyte-OPC co-cultures with a CXCL10-neutralizing antibody along with TNF-α/IL-1β. Treatment with the CXCL10-neutralizing antibody along with TNF-α/IL-1β rescued OPC proliferation and cell number in C/C astrocyte-OPC co-cultures, to a level similar to that in T/T or C/T astrocyte-OPC co-cultures treated with control IgG along with TNF-α/IL-1β (Figures 6A–6C, S7A, and S7B). Treatment with the CXCL10-neutralizing antibody along with TNF-α/IL-1β also increased OPC proliferation in T/T astrocyte-OPC co-cultures mildly (Figures S7C and S7D). Moreover, we added a CXCL10-neutralizing antibody or a control IgG to the conditioned medium of TNF-α/IL-1β-treated C/C astrocytes and applied the conditioned medium to OPCs. The CXCL10-neutralizing antibody but not a control IgG rescued OPC proliferation and O4^+^ OPC number (Figures S6G and S6H). These results together indicate that CXCL10 secreted by TNF-α/IL-1β-treated C/C astrocytes is important for inhibition of OPC proliferation.

To determine if elevated CXCL10 induction by TNF-α/IL-1β in C/C astrocytes is critical for reduced number of myelinating oligodendrocytes in astrocyte-OPC co-cultures with C/C astrocytes, we treated C/C astrocyte-OPC co-cultures on nanofibers with a CXCL10-neutralizing antibody along with TNF-α/IL-1β. Treatment with the CXCL10-neutralizing antibody along with TNF-α/IL-1β in C/C astrocyte-OPC co-cultures increased the number of MBP^+^ oligodendrocytes and the area of MBP-covered nanofibers, to a level similar to that in T/T or C/T astrocyte-OPC co-cultures treated with IgG and TNF-α/IL-1β (Figures 6D–6F). These results indicate that elevated induction of CXCL10 expression by TNF-α/IL-1β in C/C astrocytes is essential for inhibition of OPC proliferation and decreased number of oligodendrocytes.

To further determine the effect of CXCL10 induction in C/C astrocytes on myelination, we treated astrocyte-neuron-OPC co-cultures containing C/C astrocytes with the CXCL10-neutralizing antibody or a control IgG. Treatment of astrocyte-neuron-OPC co-cultures containing T/T or C/T astrocytes with IgG was included as a control. The length of MBP^+^NF^+^ axons in the co-cultures was measured as an indication of myelination. Treatment with the CXCL10-neutralizing antibody in C/C astrocyte-neuron-OPC co-cultures increased the length of MBP^+^NF^+^ axons to a level comparable with that in T/T or C/T astrocyte-neuron-OPC co-cultures treated with IgG (Figures 6G and 6H). This result indicates that elevated CXCL10 expression in C/C astrocyte-neuron-OPC co-cultures can reduce myelination and a CXCL10-neutralizing antibody can rescue this defect.

### Human brains from CLU rs11136000 C/C carriers exhibit increased expression of CLU and CXCL10 but decreased expression of MBP

**Figure 7.**
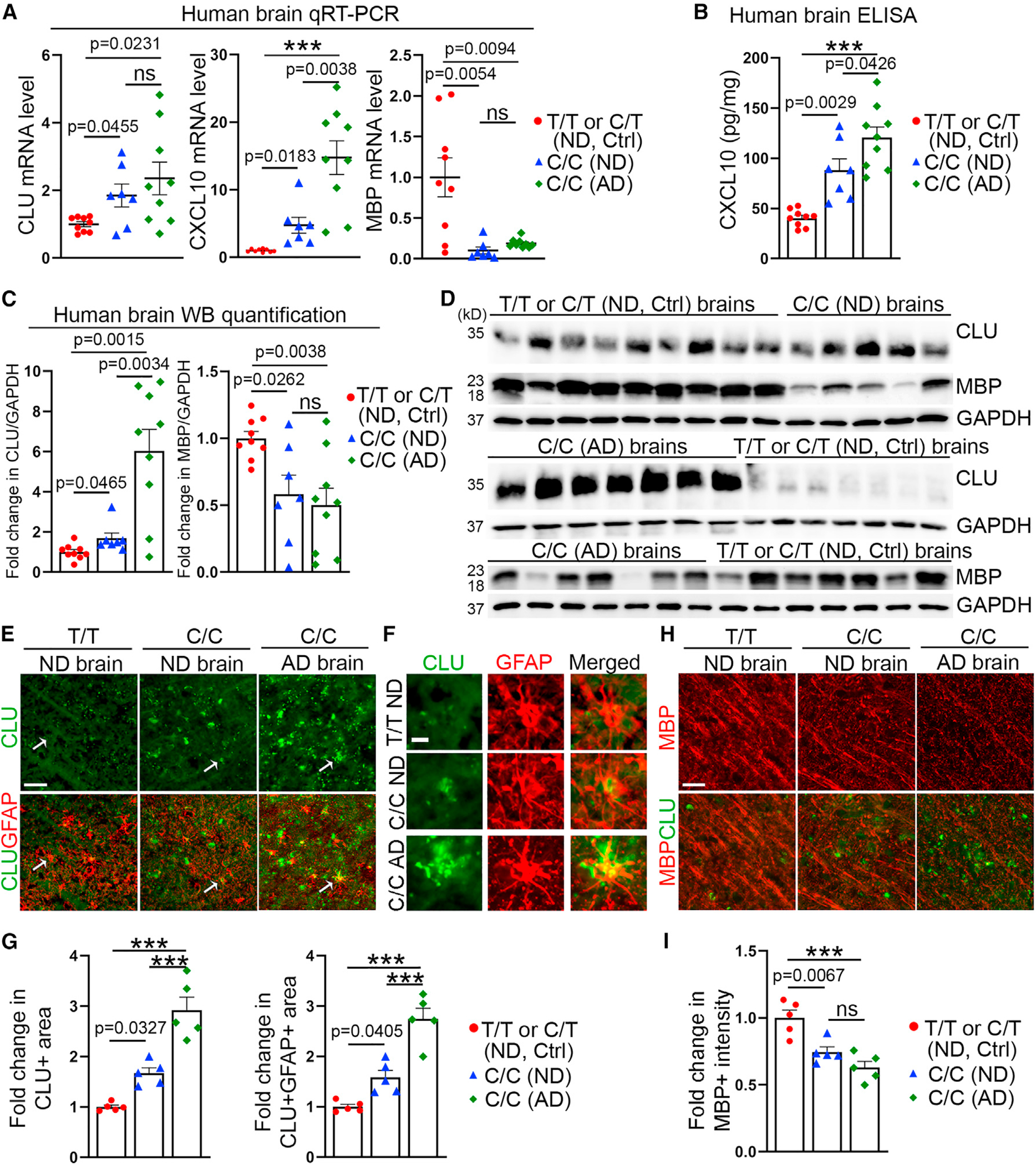
Increased CLU and CXCL10 but decreased MBP expression levels in C/C human brains (A) Elevated level of CLU and CXCL10 mRNA but reduced level of MBP mRNA in C/C vs. T/T or C/T brains revealed by qRT-PCR. (B) Elevated CXCL10 protein level in C/C vs. T/T or C/T brains revealed by ELISA. (C and D) Elevated CLU and reduced MBP protein level in C/C vs. T/T or C/T brains revealed by western blot. The same set of T/T or C/T ND brain tissues were included as controls in the two blots, with 9 lysates in blot 1 and 7 in blot 2. Because the C/C AD group had strong CLU bands, the exposure time for blot 2 was shorter than that for blot 1. The fold change (C) is relative to data in T/T or C/T ND brains. (E–G) Elevated CLU signal in C/C vs. T/T brains revealed by immunostaining. Representative images of CLU and GFAP double staining in brain tissues are shown in (E). Enlarged images of single GFAP^+^ astrocyte are shown in (F). The fold change (G) is relative to data in T/T or C/T ND brain tissues. (H and I) Reduced MBP signal in C/C vs. T/T brains revealed by immunostaining. Representative images of MBP and CLU double staining are shown in (H). The fold change (I) is relative to data in T/T or C/T ND brain tissues. Error bars are SEM of the mean. p values are indicated or labeled ***(p < 0.001), analyzed using one-way ANOVA followed by Tukey’s multiple-comparison test for (A)–(C), (G), and (I). ns, not statistically significant (p > 0.05). n = 9 subjects for T/T or C/T ND, control (Ctrl) group, 7 subjects for C/C ND group, and 9 subjects for C/C AD group for (A)–(C), n = 5 subjects per group for (G) and (I). Scale bar: 50 μm for (E) and (H) and 10 μm for (F).

To determine if elevated CLU and CXCL10 expression in C/C astrocytes occurs in human brains from CLU rs11136000 C/C carriers, we obtained brain tissues from C/C carriers, including non-demented (ND) control subjects or patients with AD, and included brain tissues from age and gender-matched T/T or C/T carrying ND subjects as controls (Table S5). Samples were collected with a mean postmortem interval (PMI) of 1.5–6 h from the frontal cortex.^70^ qRT-PCR analysis revealed an increase in CLU expression in both control and AD C/C brains, compared with that in control T/T or C/T brains (Figure 7A). We also observed elevated expression of CXCL10 in both control and AD C/C brains, compared with that in T/T or C/T brains, with more dramatic elevation in AD C/C brains than that in control C/C brains (Figures 7A–7D and S8). In contrast to elevated expression of CLU and CXCL10, we observed reduced expression of MBP, a critical component of myelin sheaths, in C/C brains (including control and AD C/C brains), compared with T/T or C/T brains (Figures 7A, 7C, and 7D). These results indicate that there is elevated expression of CLU and CXCL10 but reduced MBP expression in C/C brains.

Furthermore, we co-stained brain tissues from C/C, C/T, or T/T carriers for CLU and GFAP. Quantification revealed that both the overall CLU^+^ area and the CLU^+^GFAP^+^ area increased in C/C brains compared with that in T/T or C/T brains, with more dramatic increase in AD C/C brains than that in control C/C brains (Figures 7E, 7F, and 7G), supporting the idea that CLU expression is increased in C/C astrocytes compared with T/T or C/T astrocytes in human brains and increased in AD brains compared with that in control brains. In parallel, we stained C/C, C/T, or T/T brain tissues for MBP, a marker of myelination (Figure 7H). The MBP^+^ intensity was reduced in C/C brains compared with that in T/T or C/T brains (Figure 7I), with elevated CLU signal associated with reduced MBP signal (Figures 7G and 7I). This result indicates reduced myelination in C/C brains compared with T/T or C/T brains, consistent with the result from human brain imaging study.^64^

## DISCUSSION

In this study, we identified the most widely studied CLU SNP rs11136000 and its adjacent SNP rs1532278 and SNP rs2279590 as functional variants by CRISPR-Cas9 KO-based screen of the rs11136000 SNP and SNPs that exhibit high linkage equilibrium with it. Moreover, by switching the risk “C” or the protective “T” allele in CLU SNP rs11136000 using CRISPR-Cas9 editing, we generated isogenic iPSCs that have different alleles of the CLU rs11136000 SNP. Astrocytes derived from the isogenic iPSCs carrying the “C” or “T” alleles exhibited different CLU expression level and inflammatory response following cytokine treatment. C/C astrocytes expressed a higher level of CLU expression and exhibited elevated IFN response and CXCL10 expression level upon TNF-α/IL-1β treatment. Accordingly, C/C astrocytes inhibited OPC proliferation and myelination in astrocyte-OPC co-cultures treated with TNF-α/IL-1β.

A link between CLU and AD was established when elevated expression of CLU was detected in brains from patient with AD.^44–46^ The connection of CLU with AD was further strengthened when rs11136000, an intronic variant of CLU, was found to be associated with AD in a statistically significant manner in two independent genome-wide association studies.^13,14^ However, because rs11136000 is in strong linkage disequilibrium with a few other SNP variants in CLU, including rs1532278, rs9331896, and rs9331888,^71–73^ it remained unclear whether CLU rs11136000 is a functional variant. In this study, by knocking out CLU rs11136000 and variants that are in strong linkage disequilibrium with it, we showed that the region containing the CLU rs11136000 SNP plays an important role in the regulation of CLU expression. By switching the risk allele “C” to the protective allele “T” in CLU rs11136000 and vice versa, we demonstrated that the CLU C/C-carrying astrocytes exhibited higher CLU expression than the “T” allele-carrying (T/T or C/T) astrocytes, consistent with the prediction that higher CLU expression is associated with the “C” allele based on the expression quantitative trait loci (eQTL) data from the Genotype-Tissue Expression (GTEx) project.^74^ Thus, this study provides direct evidence that the CLU rs11136000 SNP is a functional variant that regulates CLU expression.

A previous study showed that the CLU rs11136000 T allele is associated with higher CLU expression in the temporal cortex of non-AD subjects only and the association is not statistically significant in the cerebellar tissue,^75^ indicating that the association can be brain region- and disease status-dependent. In a more recent study,^76^ an association of the CLU rs11136000 T allele and higher CLU expression was detected in the temporal cortex from the MAYO dataset. However, in this recent study,^76^ an association of the CLU rs11136000 T allele and lower CLU expression was detected in all 13 normal brain tissues from the GTEx dataset. The association between the CLU rs11136000 T allele and lower CLU expression detected in the GTEx dataset^76^ is consistent with our observation.

Of interest to us, we were able to detect different intensity of nuclear proteins binding to the “C” vs. the “T” allele-containing SNP. Differential binding to the “C” vs. the “T” allele by TDP-43 was validated using ChIP assay and CUT&RUN-qPCR in C/C vs. T/T astrocytes. TDP-43 is a DNA and RNA-binding protein that can shuttle between the nucleus and the cytoplasm and is localized primarily in the nucleus under physiological conditions.^77^ It has been shown that TDP-43 can regulate transcription, RNA splicing, export, stability, and translation.^78^ TDP-43 aggregates in cytoplasmic inclusion bodies are characteristic pathological features of amyotrophic lateral sclerosis (ALS) and frontotemporal dementia (FTD)^79,80^ and have been reported to also occur in AD.^81–83^ Although TDP-43 was initially identified as a transcriptional repressor of HIV-1 gene expression,^84^ a recent transcriptome-wide study revealed that it acts mainly as a transcriptional activator of protein-coding genes,^85^ consistent with our observation that increased binding of TDP-43 to the “C” allele is associated with elevated CLU expression. This study identifies TDP-43 as a CLU SNP-binding protein that regulates CLU expression and suggests a mechanism underlying TDP-43 contribution to the risk for AD by involving nuclear TDP-43-mediated regulation.

Increasing evidence supports the idea that defects in oligodendrocytes and myelination play important roles in the pathogenesis of AD.^7,43,86–88^ Patients with AD exhibit deficits in the white matter at multiple levels, including radiological, pathological, and molecular levels,^40,41,88–90^ reflecting demyelination status.^42^ Single-cell or single-nucleus RNA-seq analysis of the prefrontal cortex of patients with AD revealed that oligodendrocytes are among cell types with most changes in brains from patient with AD.^43,91,92^ Imaging studies have revealed that loss of white matter occurs before cognitive decline in individuals at high risk for AD,^93–95^ implying that myelin deficits could be an early events in AD pathogenesis and a key pathological element that leads to cognitive deterioration.^7^ However, mechanisms underlying myelination defects in AD remain largely unknown. In this study, we used human iPSC-derived astrocytes and OPCs co-cultured to demonstrate that TNF-α/IL-1β-induced IFN response in astrocytes could lead to inhibition of OPC proliferation and myelination.

To evaluate whether the CLU rs11136000 variant is associated with white matter deficiency, healthy young adults were imaged to evaluate their white matter integrity in an imaging study.^64^ Of particular interest, healthy young adults who carry two copies of the risk “C” allele (C/C) of CLU rs11136000 exhibited reduced white matter integrity in multiple brain regions, including those involved in AD degeneration, compared with the “T” allele carriers.^64^ However, how the CLU risk allele causes myelination defects remains largely unknown. In this study, we demonstrated that CLU C/C astrocytes exhibited more potent IFN response and released higher level of CXCL10 in response to cytokine treatment than C/T or T/T astrocytes. Elevated level of CXCL10 from C/C astrocytes or their conditioned medium reduced OPC proliferation and myelination, which could be rescued by the treatment with a CXCL10-neutralizing antibody. Our study suggests that elevated CXCL10 expression in astrocytes could represent a mechanism underlying myelination defects observed in the CLU rs11136000 risk allele carriers and patients with AD. This knowledge could help us to design more effective strategies to treat AD by targeting the IFN response or its downstream effector CXCL10 that are upstream of myelination deficits, an early event in AD pathogenesis that proceeds before cognitive decline.

### Limitations of the study

The unique advantage of the hiPSC-based disease-modeling platform is the ease of genetic engineering of iPSCs. However, hiPSCs and their derived cells are phenotypically young,^96^ therefore, it is challenging to study age-associated neurode-generative diseases using hiPSC models.^6^ To overcome this challenge, we treated hiPSC-derived astrocytes with cytokines to mimic the neuroinflammatory milieu in aged brains and brains from patients with AD, which allowed us to detect myelination defect, a phenotype detected in aged and AD brains. However, to what extent cytokine treatment can mimic the aging brain milieu remains to be determined. Moreover, using human brain tissues from ND control subjects and patients with AD, we have shown that there is elevated expression of CLU and reduced expression of MBP in brains of C/C carriers, compared with that in brains of T/T or C/T carriers. However, we included T/T or C/T ND, C/C ND, and C/C AD brains, but not T/T or C/T AD brains for the study because of tissue availability. Adding T/T or C/T AD brains may allow us to determine if the “C” or “T” allele affects CLU expression differently in the context of normal or AD brains. Nevertheless, this study allowed us to uncover a mechanism underlying reduced white matter integrity observed in the CLU rs11136000 risk “C” allele carriers, which can help us to develop AD therapies by targeting events upstream of myelination deficits.

## STAR★METHODS

Detailed methods are provided in the online version of this paper and include the following:

### RESOURCE AVAILABILITY

#### Lead contact

Further information and requests for resources and reagents should be directed to and will be fulfilled by the lead contact, Yanhong Shi (yshi@coh.org).

#### Materials availability

All unique reagents generated in this study are available from the lead contact with a completed materials transfer agreement.

#### Data and code availability

- The primary data supporting the results in this study are available within the paper and its Supplementary Information. The data-sets that support the findings of this study have been deposited in Gene Expression Omnibus (RNA-seq data) and MassIVE (mass spec data) and are publicly available as of the date of publication. Accession numbers are listed in the key resources table.
- This paper does not report original code.
- Any additional information required to reanalyze the data reported in this paper is available from the lead contact upon request.

### EXPERIMENTAL MODEL AND SUBJECT DETAILS

#### Isogenic iPSCs lines generated by CRISPR/Cas9

iPSC1-C/T (female) derived from ADRC18 fibroblast was obtained from UCI (University of California Irvine), iPSC2-C/C (female) derived from AG06869 fibroblast and have ApoE4/4 edited to ApoE3/3 and iPSC3-C/C (male) derived from AG14048 fibroblast were generated in the Shi laboratory. For gene-editing, iPSCs were transfected with the Cas9 protein and sgRNA with or without ssODN using 4D Nucleofector (Lonza). After electroporation, cells were seeded at low density onto Matrigel (1:100 diluted in DMEM/F12 medium)-coated plates and cultured in mTeSR1 medium supplemented with 10 mM Rock Inhibitor for overnight. Starting the next day, cells were cultured at 37°C, 5% CO_2_ on mTeSR1 medium.

#### Human primary astrocytes culture

Human primary astrocytes were purchased from ScienCell (Cat# 1800) and maintained at 37°C, 5% CO_2_ on Matrigel (1:100 diluted in DMEM/F12 medium)-coated tissue culture plates in astrocyte culture medium containing 1x N2, 1x B27 without Vitamin A (Life Technologies), 1X NEAA, 1X Glutamax, 10 ng/mL EGF, and 10 ng/mL FGF. These astrocytes were passaged once a week. Gender information of this product is not released by the vendor.

#### Human brain tissues

Frozen human brain tissues from non-demented (ND) control subjects or patients with AD were obtained from Banner Sun Health Research Institute. The subjects have an age range of 59-year-old and above, including 13 male and 12 female. The evaluation from Institutional Review Board of City of Hope determined these tissues from deceased subjects without identifiers do not meet the definition of human subjects research. RNAs from frozen brain tissues were isolated using Trizol. Proteins from frozen brain tissues were isolated using Pierce RIPA (Thermo Scientific).

### METHOD DETAILS

#### Generation of isogenic iPSCs using CRISPR/Cas9

Cas9 2NLS nuclease was used in this study. Guide RNAs were designed to generate DNA double-strand breaks using an online designing tool (https://www.benchling.com/crispr/). Cas9 2NLS nuclease and the oligonucleotides for sgRNA were synthesized by Synthego. For SNP KO, 2 sgRNAs were used for each SNP KO. The single-strand donor DNA (ssODN) contains C to T or T to C substitution at rs11136000 site of the human *CLU* gene. The sequences of sgRNAs and ssODNs were summarized in Table S6.

C/C or C/T iPSCs were transfected with the Cas9 protein and the ssODN using 4D Nucleofector (Lonza). After electroporation, cells were seeded at low density onto Matrigel (1:100 diluted in DMEM/F12 medium)-coated plates and cultured in mTeSR1 medium supplemented with 10 mM Rock Inhibitor for overnight. The next day, cells were fed with fresh mTeSR1 medium. Cells were maintained in mTeSR1 medium for about 10–14 days to allow colony formation from single cells. To screen for gene-corrected clones, individual colonies were manually split into 2 halves. One-half was used for genomic DNA extraction and the other half was seeded into 48-well plates for maintenance. PCR was performed to amplify the target region using genomic DNA as the template. For SNP KO, clones showed reduced size of PCR products were further analyzed by Sanger sequencing to confirm the SNP deletion. For C to T or C to C substitution, the PCR products were digested using the ApoI restriction enzyme. Clones that showed correct size of the digested products were further analyzed by Sanger sequencing to confirm the genotyping. PCR primers are listed in Table S6.

iPSC lines were assessed by G-banded karyotyping on C/C, C/T and T/T isogenic lines, and qPCR-based karyotyping on SNP KO lines. The potential off-target sites were predicted by an online tool (https://www.sanger.ac.uk/htgt/wge/find_off_targets_by_seq). The top 10 off-target sites sorted by priority on mismatches, exonic, intronic, or intergenic with sgRNAs were PCR-amplified and sequenced by Sanger sequencing. The potential off-target site sequences, the sequences of the PCR primers and the analysis results are listed in Table S3.

#### Karyotyping analysis

For G-banded karyotyping analysis, 4 wells of iPSCs in a 6-well plate with over 80% confluency were collected. G-banded karyotyping analysis was performed by the Cytogenetics Core at City of Hope. For qPCR-based karyotyping analysis, genomic DNA was extracted using PureLink Genomic DNA Mini Kit (Thermo Scientific). 300 ng genomic DNA was used for detecting the critical minimal regions of 8 most commonly mutated regions by qPCR using hPSC Genetic Analysis Kit (Stemcell Technologies) on ViiA 7 Real-Time PCR Instrument (Applied Biosystems). Data were analyzed by uploading qPCR data into online Genetic Analysis App (https://shiny.stemcell.com/ShinyApps/psc_genetic_analysis_app/), which performed statistical analysis, assisted with data interpretation, and provided visual representation of the data.

#### Differentiation of astrocytes from human iPSCs

For astrocyte differentiation, human iPSCs were first differentiated into neural progenitor cells (NPCs) by treating with 1X N2 (Life Technologies), 1X B27 (Life Technologies), 1X NEAA, 1X Glutamax, 10 ng/mL leukemia inhibitory factor (LIF; Peprotech), 0.1 μM retinoic acid (RA; Sigma-Aldrich), 4 μM CHIR99021 (Cellagen Technology), and 3 μM SB431542 (Stemgent) for 8 days.^66,97^ NPCs were seeded onto Matrigel (1:100 diluted in DMEM/F12 medium)-coated 12-well plates at 2×10^5^ cells/well and infected with the NFIA, NFIB, and SOX9-encoding lentivirus at MOI (multiplicity of infection) = 1 in NPC culture medium containing 1X N2, 1X B27, 1X NEAA, 1X Glutamax, 10 ng/mL EGF (Peprotech), 10 ng/mL FGF (Peprotech), 0.1 μM RA, 3 μM CHIR99021, and 2 μM SB43154 in the presence of 4 μg/mL polybrene (Sigma-Aldrich) one day after seeding. Infected NPCs were selected by antibiotic resistance for 10 days in NPC culture medium containing 1X N2, 1X B27, 1X NEAA, 1X Glutamax, 10 ng/mL EGF (Peprotech), 10 ng/mL FGF (Peprotech), 0.1 μM RA, 3 μM CHIR99021 and 2 μM SB431542, then switched to differentiation medium containing 2 μg/mL doxycycline, 1X N2, 1XB27, 1X NEAA, 1X Glutamax, 0.1 μM RA, and 1 μM smoothened agonist (SAG; EMD Millipore) for 14 days, then changed to 2 μg/mL doxycycline-containing PDGF medium including 1X N2, 1X B27, 1X NEAA, 1X Glutamax, 10 ng/mL PDGFAA (R&D Systems), 5 ng/mL HGF (R&D Systems), 10 ng/mL IGF-1 (R&D Systems), 10 ng/mL NT3 (EMD Millipore), 100 ng/mL Biotin (Sigma-Aldrich), 60 ng/mL T3 (Sigma-Aldrich), 1 μM cAMP (Sigma-Aldrich), and 25 μg/mL insulin (Sigma-Aldrich) for 20 days. Cells were passaged using accutase when confluent and seeded onto Matrigel (1:100 diluted in DMEM/F12 medium)-coated tissue culture plates. Then cells were cultured in astrocyte culture medium, containing 1X N2, 1X B27 without Vitamin A (Life Technologies), 1X NEAA, 1X Glutamax, 10 ng/mL EGF and 10 ng/mL FGF. These astrocytes were passaged once a week for another 2 weeks. iPSC-derived astrocytes were seeded onto Matrigel (1:100 diluted in DMEM/F12 medium)-coated 6-well plates at 1×10^5^ cells/well and maintained in astrocyte culture medium before experiments. For final maturation of astrocytes, 10 ng/mL CNTF (R&D Biosciences) was supplemented to medium containing 1X N2, 1X B27, 1X NEAA and 1X Glutamax for 1 week. The purity of the astrocytes was characterized by immunostaining for GFAP (DAKO, 1:400), S100β (Sigma-Aldrich, 1:500), or SOX9 (R&D systems, 1:200). Images were taken using Nikon Eclipse Ti2 microscope. At least 5 images of each group were used for quantification. The percentage of GFAP^+^, S100β^+^ or SOX9^+^ cells was calculated by dividing the number of GFAP, S100β or SOX9-positive cells by the number of DAPI-positive cells in the same image. The GFAP or S100β-positive cells were counted manually using the ImageJ software, the SOX9-positive cell and DAPI-positive cells were count automatically using the NIS-Elements AR Analysis 5.20.02 software.

#### Differentiation of OPCs and oligodendrocytes from human iPSCs

human iPSCs were dissociated into single cells and seeded at 1×10^5^ cells/well onto Matrigel (1:100 diluted in DMEM/F12)-coated 6-well plates in medium including 1X N2, 1X B27, 1X NEAA, 1X Glutamax 10 μM SB434542 (Stemgent), 250 nM LDN-193189 (Stemgent), and 0.1 μM RA for 8 days. From day 8–12, cells were further induced by culturing in 1X N2, 1X B27, 1X NEAA, 1X Glutamax, 0.1 μM RA and 1 μM SAG. After RA and SAG induction, pre-OPCs expressing OLIG2 and NKX2.2 markers were lifted to form spheres. The pre-OPC spheres were cultured in 0.1 μM RA and 1 μM SAG-supplemented medium for another 8 days, then switched to PDGF medium including 1X N2, 1X B27, 1X NEAA, 1X Glutamax, 10 ng/mL PDGFAA (R&D Systems), 5 ng/mL HGF (R&D Systems), 10 ng/mL IGF-1 (R&D Systems), 10 ng/mL NT3 (EMD Millipore), 100 ng/mL Biotin (Sigma-Aldrich), 60 ng/mL T3 (Sigma-Aldrich), 1 μM cAMP (Sigma-Aldrich), and 25 μg/mL insulin (Sigma-Aldrich). Ten days after switching to PDGF medium, spheres were attached onto Matrigel (1:100 diluted in DMEM/F12 medium)-coated tissue culture plates to allow OPCs to migrate out of the spheres and expand. Medium was changed every 2 days. O4^+^ OPCs could be detected by live staining using an O4-specific antibody around 30 days after sphere attachment. MBP^+^ mature oligodendrocytes emerge in another 2 weeks by culturing OPCs in glial maturation medium that has the growth factors, PDGF, IGF, HGF and NT3, withdrawn from the PDGF medium.^66,98^

#### Differentiation of neurons from human iPSCs

Human iPSCs were first differentiated into NPCs.^97^ NPCs were seeded onto Matrigel (1:100 diluted in DMEM/F12 medium)-coated 12-well plates at 2×10^5^ cells/well and infected with NGN2- and ASCL1-encoding lentivirus at MOI = 1 in NPC culture medium containing 1X N2, 1X B27, 1X NEAA, 1X Glutamax, 10 ng/mL EGF (Peprotech), 10 ng/mL FGF (Peprotech), 0.1 μM RA, 3 μM CHIR99021, and 2 μM SB43154 in the presence of 4 μg/mL polybrene (Sigma-Aldrich) one day after cell seeding. 48 h later, NPCs were subjected to antibiotic selection with 2 μg/mL puromycin in NPC culture medium. After 5 days of antibiotic selection, NPCs were seeded onto Matrigel (1:100 diluted in DMEM/F12 medium)-coated 6-well plates at 2×10^6^ cells/well and induced with neural differentiation medium containing 1X N2, 1X B27, 1X NEAA, 1X Glutamax, supplemented with 2 μg/mL doxycycline in DMEM/F12. After 4 to 5 days of induction, neurons start to emerge. The resultant neurons were cultured in neuron maintenance medium containing 1X N2, 1X B27, 100 μg/mL dibutyryl cAMP (Sigma-Aldrich), 10 ng/mL GDNF (Perprotech), and 10 ng/mL BDNF (Perprotech) in BrainPhys Neuronal Medium.

#### Cell sorting

The O4^+^ OPCs were sorted using magnetic-activated cell sorting (MACS) following manufacturer’s instruction (Miltenyi Biotech).^66^ OPCs were dissociated into single cells using accutase. These cells were incubated with O4-microbeads (10 μL antibody was used for 1×10^7^ cells in 100 μL volume) at 4°C for 15 min. Cell suspension was loaded onto LS Magnetic Column (Miltenyi Biotech) placed in the field of a magnetic MACS Separator. The O4-negative cells were washed off, while the O4-positive OPCs were retained, and eluted into a collection tube.

#### Lentiviral preparation and transduction

The NFIA, NFIB and SOX9 lentiviral vector was prepared by cloning the NFIA, NFIB and SOX9 fragment into the pLVXTP vector for lentivirus package. Lentiviruses were packaged through PsPAX2, PMD2.G and pLVXTP vectors co-transfection into HEK293T cells.^99,100^ To transduce NPCs, cells were seeded onto Matrigel (1:100 diluted in DMEM/F12 medium)-coated plates for overnight and then transduced with lentivirus in the presence of 4 μg/mL polybrene (AmericanBio) for 24 h. Virus-containing medium was replaced with fresh NPC culture medium 24 h later. Antibiotic selection was started 2 days after virus infection.

The sequences of the control shRNA (shC) and the shRNAs for TDP-43 (shTDP-43-2, and shTDP-43-3) were cloned into the pHIV7-GFP lentiviral vector for lentiviral packaging. The target sequences for the shRNAs are as follows: shTDP-43-2: 5′-GGA AAC AAT CAA GGT AGT AAT-3′, shTDP-43-3: 5′-CTC TAA TTC TGG TGC AGC AAT-3’. To transduce iPSC-derived astrocytes, cells were seeded onto Matrigel (1:100 diluted in DMEM/F12 medium)-coated plates for overnight and then transduced with lentivirus in the presence of 4 μg/mL polybrene for 24 h. Virus-containing medium was replaced with fresh astrocyte culture medium 24 h later.

#### Immunohistochemistry

Cells were seeded on Matrigel (1:100 diluted in DMEM/F12 medium)-coated black 96-well plates (Greiner Bio-One, iBidi) for tissue culture with flat and clear bottom for microscopy. Cells were fixed with 4% paraformaldehyde (PFA) for 15 min, permeabilized with 0.1% Triton X-100 for 1 h and blocked with 5% donkey serum for 1 h at room temperature (RT). Cells were then incubated with primary antibody diluted in PBS containing 0.1% Triton X-100 and 5% donkey serum and incubated for overnight at 4°C. On the following day, cells were incubated with the relevant secondary antibody diluted at 1:500 in PBS for 1 h at RT. Cells were counterstained with DAPI before mounting.

For astrocytes characterization, cells were stained for GFAP (DAKO, 1:400), S100β (Sigma-Aldrich, 1:500), or SOX9 (R&D systems, 1:200).

For EdU staining (proliferation assay), we followed manufacturer’s instruction (Invitrogen). Briefly, astrocytes, OPCs or astrocytes-OPCs co-culture cells were incubated with 10 μM EdU for 5 h, and then fixed with 4% PFA for EdU staining. Then cells were incubated with EdU reaction cocktail for 30 min. After PBS wash, astrocytes were stained for SOX9 (R&D systems, 1:200), OPCs were stained for OLIG2 (EMD Millipore, 1:200) or SOX10 (R&D systems, 1:200, 5 μg/ml). Cells were counterstained with DAPI before mounting.

For human brain staining, frozen brain cortex tissues of healthy controls and patients with AD were fixed with 4% PFA at 4°C for overnight, followed by 30% sucrose incubation at 4°C for overnight. Then brain tissues were embedded in OCT compound and sectioned at a thickness of 16 μm using Leica CM3050S. For antigen retrieval, slides were immersed in citrate buffer, pH 6.0 (Sigma), in a microwaveable container and heated in microwave. The microwave heating was stopped when the citrate buffer was boiled for 5 s. The container with the slides was taken out from microwave and cooled to room temperature. The boiling and cooling process was repeated once. Then the slides were washed with water. To reduce the background autofluorescence, a quenching procedure was used.^101,102^ Brain sections were incubated with 0.3% KMnO_4_ (w/v) for 5 min, washed in water, then treated with 1% K_2_S_2_O_5_ and 1% oxalic acid until the brown color was removed from the tissues. Slides were washed with water and the quenching procedure was repeated once. After washing, brain tissue slides were blocked with 5% donkey serum for 1 h at room temperature and stained for CLU (Santa Cruz, 1:1000, 200 ng/mL), MBP (Millipore, 1:200) or GFAP (DAKO, 1:400). For CLU staining, SuperBoost kit (Invitrogen) was used to enhance the signals. Antibodies used in this study are listed in key resources table.

#### Astrocyte-OPC co-culture

Black 96-well plates for tissue culture with flat and clear bottom for microscopy were purchased from Greiner Bio-One. Mature astrocytes (treated with 10 ng/mL CNTF for 1 week) were seeded at 8×10^3^ cells per well on Matrigel (1:100 diluted in DMEM/F12 medium)-coated 96-well plates. The day after astrocyte seeding, O4^+^ OPCs sorted by MACS were seeded at 5×10^3^ cells per well onto astrocytes in Matrigel-coated 96-well plates. Co-cultured cells were maintained in PDGF medium with or without treatment. For treatment experiments, co-cultures were subjected to treatment with 25 ng/mL TNFα and 10 ng/mL IL1β or the combination of TNFα/IL1β with vehicle, 100 ng/mL human CLU protein, 1 μg/mL IgG, or 1 μg/mL CXCL10 neutralizing antibody (as indicated in relevant figure legends) the day after OPC seeding. The treatment is continued until cells were harvested.

For O4^+^ cell number quantification, OPCs were co-cultured with astrocytes for 6 days. On day 6 of co-culture, cells were fixed and stained for O4 (Sigma-Aldrich, 1:200, 1 μg/ml) and GFAP (DAKO, 1:400).

For proliferation assay, OPCs were co-cultured with astrocytes for 2 days. On day 2 of co-culture, cells were stained for EdU and OLIG2 (EMD Millipore, 1:200) or SOX10 (R&D systems, 1:200, 5 μg/ml).

For apoptosis assay, OPCs were co-cultured with astrocytes and co-cultures were subjected to treatment with 25 ng/mL TNFα and 10 ng/mL IL1β for 1 day. On day 2 of co-culture, cells were stained for cleaved caspase 3 (Cell Signaling Technology, 1:400) and SOX10 (R&D systems, 1:200, 5 μg/ml).

Images were taken using Nikon Eclipse Ti2 microscope. 5 images per well were taken for quantification. The number of samples and the number of independent experiments are indicated in the figure legends. The EdU, OLIG2 and SOX10-positive cells were counted automatically using the NIS-Elements AR Analysis 5.20.02 software. The O4 and Cleaved caspase3-positive cells were counted manually using the ImageJ software. The calculation of the fold change in the O4^+^ cell number (#) is described in the figure legends. %EdU^+^OLIG2^+^ cells was calculated by dividing the number of EdU and OLIG2-double-positive cells by the number of the OLIG2-positive cells. %EdU^+^SOX10^+^ cells was calculated by dividing the EdU and SOX10-double-positive cells by the number of SOX10-positive cells. %Cleaved Cas3^−^SOX10^+^ cells was calculated by dividing the number of SOX10-positive but cleaved caspase 3-negative cells by the number of SOX10-positive cells.

#### Conditioned medium preparation and neutralizing antibody treatment

T/T, C/T, or C/C astrocytes were seeded at 1.5×10^5^ cells/well in a Matrigel (1:100 diluted in DMEM/F12 medium)-coated 6-well plate. Cells were conditioned in PDGF medium with or without 25 ng/mL TNFα and 10 ng/mL IL1β treatment for 24 or 48 h. Conditioned medium was collected and centrifuged at 200× g for 10 min and supernatant was filtered through 0.22 μm filter to remove residual cells and cell debris. O4^+^ OPCs were sorted using MACS with an O4 antibody and eluted with astrocyte conditioned medium. OPCs were seeded at 5×10^3^ cells/well onto Matrigel (1:100 diluted in DMEM/F12 medium)-coated black 96-well plates and cultured in astrocyte conditioned medium. For neutralizing antibody treatment, cells were cultured in astrocyte conditioned medium with 1 μg/mL CXCL10 neutralizing antibody or the corresponding control IgG (as indicated in relevant figure legends).

For O4^+^ cell number quantification, OPCs were cultured in astrocyte conditioned medium for 6 days. On day 6 of culture, cells were fixed and stained for O4 (Sigma-Aldrich, 1:200, 1 μg/ml) and GFAP (DAKO, 1:400).

For proliferation assay, OPCs were cultured in astrocyte conditioned medium for 2 days. On day 2 of conditioned medium treatment, cells were stained for EdU and OLIG2 (EMD Millipore, 1:200).

Images were taken using Nikon Eclipse Ti2 microscope.5 images per well were taken for quantification. The number of samples and the number of independent experiments are indicated in the figure legends. The EdU- and OLIG2-positive cells were counted automatically using the NIS-Elements AR Analysis 5.20.02 software. The calculation of the fold change in the O4^+^ cell number (#) is described in the figure legends. %EdU^+^OLIG2^+^ cells were calculated by dividing the number of EdU and OLIG2-double-positive cells by the number of the OLIG2-positive cells.

#### 3D nanofiber myelination assay

Eight-chamber slides aligned with 700 nm diameter electrospun polycaprolactone (PCL) nanofibers were purchased from Nanofiber Solutions. Nanofiber 96-well plates with aligned nanofibers were purchased from MilliporeSigma. Matrigel (1:100 diluted in DMEM/F12 medium) was used to coat nanofibers at 37°C for 3 days. Mature astrocytes were seeded at 1×10^4^ cells (100 μL cells at 1×10^5^ cells/ml) per well in 96-well plates and 2.5×10^4^ cells (250 μL cells at 1×10^5^ cells/ml) per well in eight-chamber slides. The next day, 50 mL medium per well was removed from 96-well plates and 125 μL medium per well was removed from eight-chamber slides. Then O4^+^ OPCs sorted by MACS were seeded at 8×10^3^ cells (100 μL cells at 8×10^4^ cells/ml) per well in astrocyte-containing 96-well plates and 2×10^4^ cells (250 μL cells at 8×10^4^ cells/ml) per well in astrocyte-containing eight-chamber slides, with a total of 150 μL medium per well was used in 96-well plates and 375 μL medium per well in eight-chamber slides during co-culture. For treatment experiments, co-cultures were treated with 12.5 ng/mL TNFα and 5 ng/mL IL1β or the combination of TNFα/IL1β with vehicle, 100 ng/mL human CLU protein, 1 μg/mL control IgG, or 1 μg/mL CXCL10 neutralizing antibody (as indicated in relevant figure legends) 2 days after seeding. The treatment was continued until cells were harvested. Cells were cultured in PDGF medium for 5 days with medium change every 2 days to maintain OPCs at the progenitor state, and then switched to glial maturation medium for 2 weeks with medium change every 2 days to allow OPCs to differentiate into mature oligodendrocytes; or directly cultured in glial maturation medium for 20 days with medium change every 2 days. After co-culturing, cells were fixed with 4% PFA and stained for MBP (Millipore, 1:200), GFAP (DAKO, 1:400), and cleaved caspase 3 (Cell Signaling Technology, 1:400).

Images were taken using Nikon Eclipse Ti2 microscope or Zeiss LSM 700 confocal microscope. 5 images per well were taken for quantification of MBP^+^ cell number and MBP^+^ area. The MBP- and cleaved caspase 3-positive cells were counted manually using the ImageJ software. The MBP-positive area was determined automatically using the NIS-Elements AR Analysis 5.20.02 software. Calculation of the fold change in MBP^+^ cell number (#) and MBP^+^ area is described in the figure legends. %Cleaved Cas3^−^MBP^+^ cells was calculated by dividing the number of MBP-positive but cleaved caspase 3-negative cells by the number of MBP-positive cells.

#### Astrocyte-neuron-OPC co-culture

Black 96-well plates for tissue culture with flat and clear bottom for microscopy were purchased from iBidi. Human iPSC-derived neurons were seeded at 1.5×10^5^ per well in Matrigel (1:100 diluted in DMEM/F12 medium)-coated 96-well plates and maintained in neuron maintenance medium to maintain neuronal cells for 5 days. On day 6, mature astrocytes were seeded at 1×10^4^ cells per well on neurons. At day 7, O4^+^ OPCs sorted by MACS were seeded at 8×10^3^ cells per well. 200 μL medium per well was used in 96-well plates during co-culture. Cells were cultured in PDGF medium for 5 days with half medium change every 2 days to maintain OPCs (in the co-culture) at the progenitor state, and then switched to glial maturation medium for 2 weeks with half medium change every 2 days to allow OPCs to differentiate into mature oligodendrocytes. Co-cultures were treated with 0.5 μg/mL control IgG or 0.5 μg/mL CXCL10 neutralizing antibody (as indicated in relevant figure legends) 2 days after seeding. The treatment continued until cells were harvested. After 20 days of co-culture, cells were fixed and stained for MBP (Millipore, 1:200) and Neurofilament 200 (Sigma-Aldrich, 1:200).

Images were taken using Nikon Eclipse Ti2 microscope or Zeiss LSM 700 confocal microscope. 3D images were generated using Zen Blue edition. 5 images per well were taken for quantification of the MBP^+^NF^+^ axon length. MBP^+^NF^+^ axon length was measured manually by Fiji software. Calculation of the fold change in the MBP^+^NF^+^ axon length is described in the figure legends.

#### RNA-seq

RNA was isolated by Trizol (Thermo Fisher).^103–105^ RNA quality control and subsequent library construction and poly (A) RNA-seq were performed by the Integrative Genomics Core at City of Hope. RNA-Seq reads were aligned against the human genome (hg19) using TopHat2.^106^ Read counts were quantified using htseq-count (version 0.6.0)^107^ with UCSC known gene annotations (TxDb. Hsapiens.UCSC. hg19.knownGene).^108^ Aligned reads were counted using GenomicRanges.^109^ Genes were filtered to only include transcripts with RPKM values greater than 0.1 (after a rounded log2-transformation) in at least 50% of samples. Genes smaller than 150 bp were removed prior to differential expression analysis. Log2(RPKM +0.1) expression values were used for visualization and fold-change calculations. Separate comparisons were performed for the 2 pairs of isogenic astrocyte lines. To identify genes that were differentially expressed in C/C astrocytes vs. T/T or C/T astrocytes, we subjected 2 isogenic astrocyte pairs (AS1: C/C vs. C/T, and AS2: C/C vs. T/T) to RNA-seq, in which AS1-C/C and AS2-C/C contain only the risk C allele, while AS1-C/T and AS2-T/T are carriers of the protective T allele. The pathway analysis was performed using genes that are differentially expressed in C/C astrocytes compared to that in T/T or C/T astrocytes. To determine genes with varied expression between C/C and T/T or C/T astrocytes, we used a 2-variable model in DESeq2. p values were calculated using DESeq2,^110^ which were used to calculate the False Discovery Rate (FDR).^111^ Differentially expressed genes (DEG) were defined as FDR <0.05 and absolute value of fold change >1.5. Signaling pathway analysis was performed using HALLMARK analysis. Gene Ontology (GO)^112^ enrichment was performed using goseq.^113^

#### qRT-PCR

Total RNA was extracted using Trizol. Complementary DNA was reverse transcribed from 1 μg total RNA using Tetro cDNA Synthesis kit (BioLINE). Primer sequences are listed in Table S7 qRT-PCR was performed using SYBR Green Master Mix (Thermo Scientific) on the Step One Plus Real-Time PCR Instrument (Applied Biosystems). GAPDH was used as the reference gene. Data was analyzed using 2^−ΔCt^ method and normalized to control group in each run.

#### Mass spectrometry

DNA binding proteins were denatured by addition SDS to a final concentration of 5%. Then proteins were reduced and alkylated with 10 mM tris(2-carboxyethyl) phosphine and 30 mM iodoacetamide. Afterward, proteins were precipitated in suspension traps (S-Trap micro, Protifi), and sample clean-up was performed according to manufacturer’s instructions. Proteins were digested in the S-Trap in presence of 2 μg Trypsin/Lys-C (Promega) for overnight at 37°C. Eluted peptides were dried and resuspended in 1% formic acid. Mass spectrometry was performed on an orbitrap Fusion Tribrid instrument (Thermo Fisher) equipped with an Easy-nLC 1000 HPLC system, a 75 μm by 2 cm PepMap C18 trapping column, a 75 μm by 50 cm PepMap RSLC C18 analytical column, and an Easy-Spray ion source. Peptides were separated by 1-h gradient with 0.1% formic acid and acetonitrile (3–30%). Precursor ion scans were acquired in the orbitrap and CID fragments were acquired in the linear ion trap. Data analysis was performed using MaxQuant software (version 1.6.17.0) with the human Uniprot reference proteome database (downloaded on 9/8/21).

#### ChIP-qPCR assay

For each ChIP reaction, 2×10^6^ cells were used. Chromatin immunoprecipitation was performed using the Magna ChIP A/G Chromatin Immunoprecipitation kit following manufacturer’s instruction (Sigma) with minor adjustments. Briefly, cells were incubated with dimethyl 3,3′ dithiobispropionimidate-HCl (DTBP) (Thermo Scientific) solution (5 mM) in dark at room temperature for 10 min, then treated with 1% formaldehyde at room temperature for 10 min.^114–116^ Then nuclear extracts were collected using NE buffer prepared from the kit. Nuclear extracts were sonicated to generate ~200–500 bp DNA fragments. Nuclear extracts, antibodies, and Protein A/G Magnetic Beads were incubated together at 4°C for overnight, after washes with low salt, high salt, LiCl and TE buffers, bound materials were eluted with the elution buffer. Immunoprecipitated samples were quantified using qPCR. Bound DNA was presented as the percentage of input DNA. Antibodies used for ChIP assays including TDP-43 (Proteintech, 5μg per reaction), Galectin-7 (R&D system, 5μg per reaction) and PCBP2 (Santa Cruz, 5μg per reaction) were listed in key resources table. The primer pair used for ChIP-qPCR of the CLU SNP1 includes forward primer 5′-GGC TGC AGA CTC CCT GAA TC-3′ and reverse primer 5′-GCA AGG GCC CGT TAG AGA AT-3.

#### CUT&RUN-qPCR assay

For each CUT&RUN reaction, 5×10^4^ cells were used. CUT&RUN was performed using the CUT&RUN assay kit following manufacturer’s instruction (Cell Signaling Technology). Briefly, cells were dissociated with accutase. The cell suspension was centrifuged at 600 × g for 3 min at room temperature and the supernatant was removed. Cell pellets were washed 3 times with 1x wash buffer. Then cell pellets were resuspended in Digitonin buffer (prepared using the material from the kit) and incubated with primary antibodies and activated Concanavalin A Magnetic Beads at 4°C for overnight. After washing with Digitonin buffer once, bound materials were treated with pAG-MNase at 4°C for 1 h. Then the pAG-MNase was activated by adding Calcium Chloride to samples and incubated with samples at 4°C for 30 min. To stop the reaction, the stop buffer with spike-in DNA prepared from the kit was added to samples and incubated with samples at 37°C for 10 min. DNA on bound materials were collected and purified using spin columns. Samples were quantified using qPCR. Bound DNA was normalized with spike-in DNA and presented as the percentage of input DNA. TDP-43 antibody (Proteintech, 5μg per reaction) was used for the CUT&RUN assay. The primers used for CUT&RUN-qPCR of the CLU SNP1 include forward primer 5′-GGC TGC AGA CTC CCT GAA TC-3′ and reverse primer 5′-GCA AGG GCC CGT TAG AGA AT-3′.

#### Western blot analysis

Cell lysates were extracted using Pierce RIPA buffer. Protein concentration was measured using BCA assay kit (Thermo Scientific). 5 μg proteins were loaded for TDP43 (Proteintech, 1:2000, 300 ng/mL) detection and 20 μg protein for CLU (Cell Signaling Technology, 1:1000), CLU (Santa Cruz, 1:500), p-STAT1 (Cell Signaling Technology, 1:1000), STAT1(Cell Signaling Technology, 1:1000) and MBP (Millipore, 1:1000) detection. Western blot membranes were developed using ECL Select kit or ECL prime kit (GE Healthcare) and imaged using ChemiDoc Imaging System (Bio-Rad). Image Lab was used for Western blot quantification. The uncropped original scans are included at the end of the manuscript. Antibodies used in this study are listed in key resources table.

#### ELISA

To detect CLU level in astrocyte medium by ELISA, astrocytes were seeded at 1×10^5^ cells/well in a Matrigel (1:100 diluted in DMEM/sF12 medium)-coated 24-well plate. Cells were cultured in astrocyte culture medium for 24 h. Medium was collected and centrifuged at 200× g for 10 min and supernatant was filtered through 0.22 μm filter to eliminate cells and cell debris. ELISA was performed with filtered supernatant according to manufacturer’s instruction (R&D Systems).

To detect CXCL10 level in astrocyte medium by ELISA, astrocytes were seeded at 1×10^5^ cells/well in a Matrigel (1:100 diluted in DMEM/F12 medium)-coated 24-well plate. Cells were treated with 25 ng/mL TNFα and 10 ng/mL IL1β in astrocyte culture medium the day after seeding for 24 h. Medium was collected and centrifuged at 200× g for 10 min and supernatant was filtered through 0.22 μm filter to eliminate cells and cell debris. ELISA was performed with filtered supernatant according to manufacturer’s instruction (MilliporeSigma).

To detect CXCL10 level in human brain tissues by ELISA, tissue lysates were extracted using Pierce RIPA buffer. Protein concentration was measured using BCA assay kit. ELISA was performed using 100 μg frontal cortex tissue lysates according to manufacturer’s instruction (MilliporeSigma), CXCL10 concentration was normalized to the amount of protein in each sample.

#### Luciferase reporter assay

The reporter plasmids TK-SNP1-luc and TK-SNP1X3-luc were generated by cloning one copy of the 31 bp sequences around C/C rs11136000 or three copies of the 31 bp sequences around C/C rs11136000 into the TK-luc vector. The pLVX-Puro-TDP-43-WT (Addgene, Cat#133753) and pLX313-Renilla luciferase (Addgene, Cat#118016) were purchase from Addgene. HEK293T cells were co-transfected with the firefly luciferase reporter vector (SNP1-luc or SNP1X3-luc), a Renilla luciferase reporter vector, and a GFP (control) or TDP43-encoding vector using Lipofectamine 3000. After 48 h, cells were assayed using a Dual-Luciferase Reporter Assay System (Promega). The Renilla luciferase reporter was included as a control to normalize for transfection efficiency. Relative luciferase activity was calculated as the firefly luciferase activity divided by the Renilla luciferase activity, and then normalized to the control GFP group.

#### Mycoplasma test

All cell culture samples were monitored by mycoplasma test at least once a month using MycoAlert PLUS Mycoplasma Detection Kit (Lonza). 500 μL culture medium was harvested and centrifuged at 200× g for 5 min to eliminate cell debris. 100 μL medium was taken for 2 reactions with reagents provided in the kit. The result was determined by luminescence reading. All cellular samples used in this study are mycoplasma negative.

### QUANTIFICATION AND STATISTICAL ANALYSIS

Statistical analysis was performed using Graphpad Prism Version 9 by unpaired two-tailed student’s t test or ANOVA as reported in each figure legend. When comparing two experimental groups, unpaired two-tailed student’s t test was used. When comparing multiple experimental groups, data was analyzed using one-way ANOVA followed by Tukey’s multiple comparison test or unpaired t test with Welch’s correction or two-way ANOVA followed by Bonferroni’s multiple comparisons test when ANOVA has p < 0.05. For all tests, p values were presented as the actual p value or ***(p < 0.001). Error bar stands for SEM if not stated otherwise. Statistical details of each experiment are described in relevant figure legends.

## Supplementary Material

1

2

3

## Figures and Tables

**KEY RESOURCES TABLE:** 

REAGENT or RESOURCE	SOURCE	IDENTIFIER

Antibodies		

Rabbit polyclonal anti-GFAP	DAKO	Cat# N1506; RRID: AB_10013482
Mouse monoclonal anti-S100b	Sigma-Aldrich	Cat# S2532; RRID: AB_477499
Rabbit polyclonal anti-OLIG2 EMD	Millipore	Cat# AB9610; RRID: AB_570666
Mouse monoclonal IgM anti-O4	Sigma-Aldrich	Cat# O7139; RRID: AB_477662
Anti-O4 MicroBeads	Miltenyi Biotec	Cat# 130-096-670; RRID: AB_2847907
Goat polyclonal anti-SOX10	R&D systems	Cat# AF2864; RRID: AB_442208
Rat monoclonal anti-MBP	Millipore	Cat# MAB386; RRID: AB_94975
Rabbit polyclonal anti-Cleaved Caspase-3	Cell Signaling Technology	Cat# 9661; RRID: AB_2341188
Rabbit polyclonal anti-STAT1	Santa Cruz	Cat# sc-346; RRID: AB_632435
Rabbit monoclonal anti-Phospho-STAT1	Cell Signaling Technology	Cat# 9167; RRID: AB_561284
Rabbit monoclonal anti-CLUSTERIN	Cell Signaling Technology	Cat# 34642; RRID: AB_2799057
Mouse monoclonal anti-CLUSTERIN	Santa Cruz	Cat# sc-5289; RRID: AB_673566
Mouse monoclonal anti-CXCL10	R&D systems	Cat# MAB266; RRID: AB_2261309
Rabbit polyclonal anti-TDP-43	Proteintech	Cat# 10782-2-AP; RRID: AB_615042
Goat polyclonal anti-Galectin-7	R&D systems	Cat# AF1339; RRID: AB_2297076
Mouse monoclonal anti-hnRNP E2	Santa Cruz	Cat# sc-101136; RRID: AB_1124684
Rabbit polyclonal anti-Neurofilament 200	Sigma-Aldrich	Cat# N4142; RRID: AB_477272
Goat polyclonal anti-SOX9	R&D systems	Cat# AF3075; RRID: AB_2194160

Biological samples		

Human brain tissue	Banner Sun Health Research Institute	www.brainandbodydonationprogram.org

Chemicals, peptides, and recombinant proteins		

DMEM/F12	GIBCO	Cat# 11330-032
Matrigel	Corning	Cat# CB40230
mTeSR1	Stem Cell Technologies	Cat# 85850
Y-27632	Reprocell	Cat# 04-0012-10
Accutase	Sigma-Aldrich	Cat# A6964
N2	Life Technologies	Cat# 17502048
B27	Life Technologies	Cat# 12587010
GlutaMax	GIBCO	Cat# 35050079
NEAA	Thermo Fisher Scientific	Cat# 11140076
CHIR99021	Cellagen Technology	Cat# C2477-50
SB431542	Stemgent	Cat# 04-0010
LDN-193189	Stemgent	Cat# 04-0074
Smoothened agonist (SAG)	EMD Millipore	Cat# 566660
bFGF	PeproTech	Cat# 100-18B
EGF	PeproTech	Cat# 100-15
Retinoic acid	Sigma-Aldrich	Cat# R2625
CNTF	R&D systems	Cat# 257-NT-050
PDGFAA	R&D systems	Cat# 221-AA-050
IGF-1	R&D systems	Cat# 291-G1-200
HGF	R&D systems	Cat# 294-HG-025
NT3	EMD Millipore	Cat# GF031
3,3’,5-Triiodo-L-thyronine (T3)	Sigma-Aldrich	Cat# T2877
Biotin	Sigma-Aldrich	Cat# 4639
Dibutyryl-cAMP	Sigma-Aldrich	Cat# D0627
L-Ascorbic acid	Sigma-Aldrich	Cat# A4403
Inuslin	Sigma-Aldrich	Cat# I9278
TGFα	Stemcell Techology	Cat# 78157.1
IL1 β	R&D systems	Cat# 201-LB-005
Human Clusterin Protein	R&D Systems	Cat# 2937-HS-050

Critical commercial assays		

Tetro cDNA Synthesis kit	BioLINE	Cat# Bio-65043
SYBR Green Master Mix	Thermo Scientific	Cat# F416L
P3 4D nucleofection kit	Lonza	Cat# V4XP-3024
Human Clusterin Quantikine ELISA Kit	R&D Systems	Cat# DCLU00
HUMAN IP-10/CXCL10 ELISA KIT	MilliporeSigma	Cat# RAB0119
Click-iT^™^ Plus EdU Cell Proliferation Kit	Invitrogen	Cat# C10637
Magna ChIP^™^ A/G Chromatin Immunoprecipitation Kit	Sigma	Cat# 17-10085
CUT&RUN Assay Kit	Cell Signaling Technology	Cat# 86652
Dual-Luciferase Reporter Assay System	Promega	Cat# E1910
Alexa Fluor^™^ 488 Tyramide SuperBoost^™^ Kit, goat anti-mouse IgG	Invitrogen	Cat# B40912
hPSC Genetic Analysis Kit	Stemcell Techology	Cat# 07550
PureLink^™^ Genomic DNA Mini Kit	Invitrogen	Cat# K182001

Deposited data		

Mass spectrometry analysis of differential binding to the C vs. T allele by nuclear proteins	This paper	MassIVE: MSV000088660
RNA-seq analysis of C/C vs. T/T or C/T astrocytes	This paper	GEO: GSE193218

Experimental models: Cell lines		

ADRC18 fibroblast	UCI-ADRC	N/A
AG14048 fibroblasts	Coriell	Cat# AG14048
AG06869 fibroblasts	Coriell	Cat# AG06869
Human primary astrocytes	ScienCell	Cat# 1800

Oligonucleotides		

See Tables S6 and S7 for details	N/A	N/A

Software and algorithms		

NIS-Elements AR	Nikon	RRID: SCR_014329
ZEN 3.1	Carl Zeiss	RRID:SCR_013672
Image Lab Software	Bio-rad	RRID:SCR_014210
Fiji (ImageJ)	Fiji (ImageJ)	RRID: SCR_002285
Graphpad Prism 9	Graphpad Software	RRID: SCR_002798
Biorender	Biorender	RRID:SCR_018361

## Highlights

- The CLU rs11136000 SNP is identified as a functional variant
- C/C astrocytes exhibit elevated CLU expression and inflammatory response
- TDP-43 preferentially binds to the “C” allele to promote CLU expression
- Cytokine-treated C/C astrocytes inhibit OPC proliferation and myelination

## INCLUSION AND DIVERSITY

We support inclusive, diverse, and equitable conduct of research.
