# Supplementary material for: Astrocytic response mediated by the CLU risk allele inhibits OPC proliferation and myelination in a human iPSC model
Source: Cell Rep. Author manuscript; Available in PMC 2023 Sep 20. (PMC10510531; doi:10.1016/j.celrep.2023.112841)
Supplement: 1 [file NIHMS1928234-supplement-1.pdf]

Cell Reports, Volume *42*

Supplemental information

Astrocytic response mediated by the CLU risk allele inhibits OPC proliferation and myelination in a human iPSC model

Zhenqing Liu, Jianfei Chao, Cheng Wang, Guihua Sun, Daniel Roeth, Wei Liu, Xianwei Chen, Li Li, E Tian, Lizhao Feng, Hayk Davtyan, Mathew Blurton-Jones, Markus Kalkum, and Yanhong Shi

Cell Reports, Volume ■ ■

Supplemental information

Astrocytic response mediated by the CLU risk allele inhibits OPC proliferation and myelination in a human iPSC model

Zhenqing Liu, Jianfei Chao, Cheng Wang, Guihua Sun, Daniel Roeth, Wei Liu, Xianwei Chen, Li Li, E. Tian, Lizhao Feng, Hayk Davtyan, Mathew Blurton-Jones, Markus Kalkum, and Yanhong Shi

Supplementary Figures and Legends

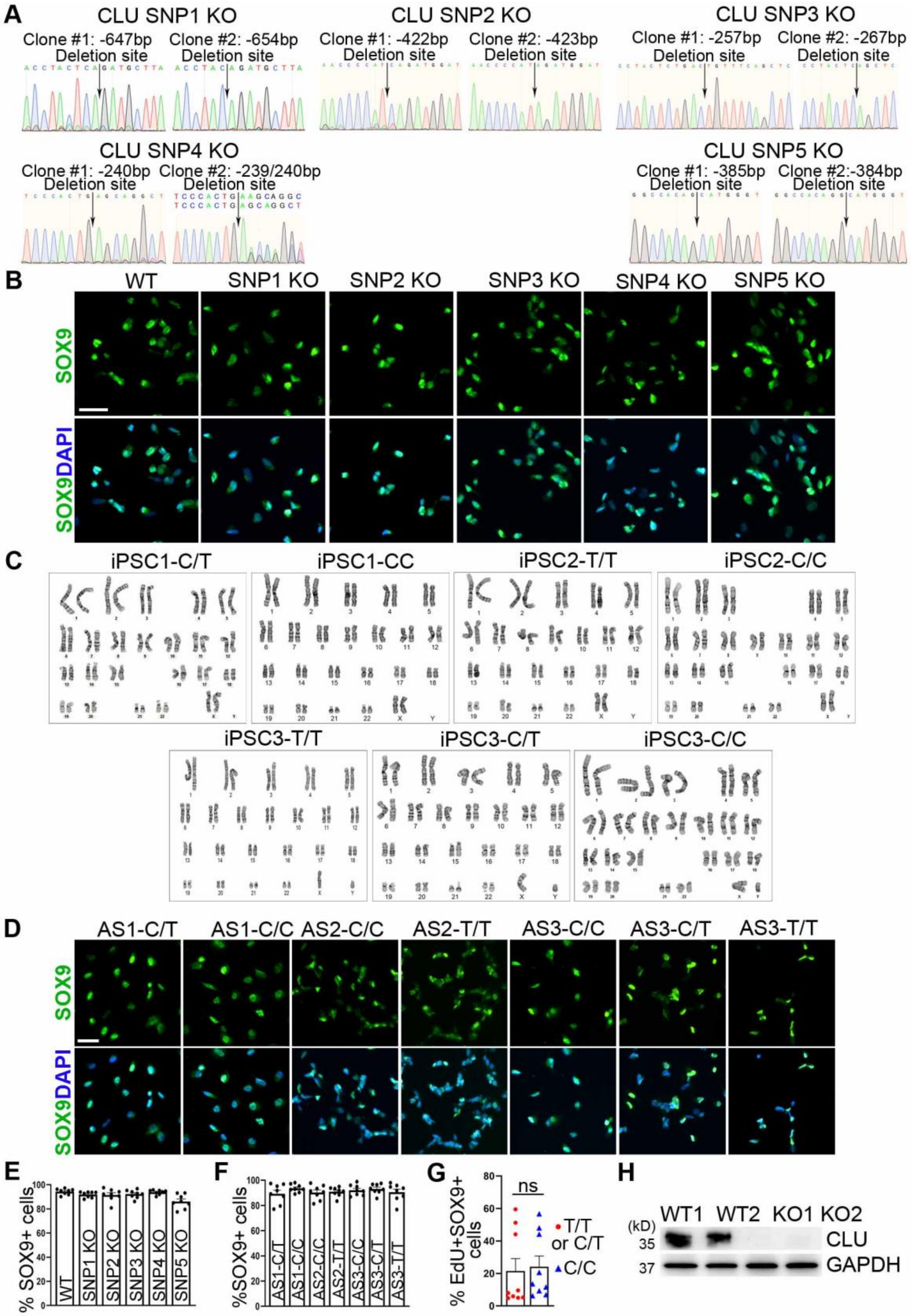

Fig. S1 Characterization of CLU SNP KO or SNP1 C/C, C/T or T/T astrocytes, related to Fig 1, 2.

(A) DNA sequence showing deletion of the CLU SNP1, SNP2, SNP3, SNP4 and SNP5 region in iPSCs.

(B) The WT and CLU SNP KO astrocytes express the astrocyte marker SOX9 as revealed by immunostaining.

(C) The CLU C/C, C/T, or T/T iPSCs exhibit normal karyotype as revealed by G-banding karyotyping.

(D) The CLU C/C, C/T, or T/T astrocytes expressed the astrocyte marker SOX9 as revealed by immunostaining.

(E) The purity of the WT or SNP KO astrocytes determined by the percentage of $SOX9^+$ cells (% $SOX9^+$ cells) based on staining images.

(F) The purity of the CLU C/C, C/T, or T/T astrocytes determined by the percentage of $SOX9^+$ cells (% $SOX9^+$ cells) based on staining images.

(G) T/T or C/T and C/C astrocyte proliferation determined by the percentage of EdU^+SOX9^+ cells.

(H) The specificity of the CLU antibody is shown by Western blot using wildtype (WT) and CLU knockout (KO) iPSC-derived brain organoids. WT1 and WT2 represent 2 organoids from the WT group and KO1 and KO2 represent 2 organoids from the KO group.

Error bars are SEM of the mean. p values are indicated in the graphs, analyzed by two-tailed student's t-test. ns: not statistically significant ($p>0.05$). n=8 images per group for (E, F), n=9 independent experiments for (G). Scale bar: 50 µm for (B, D).

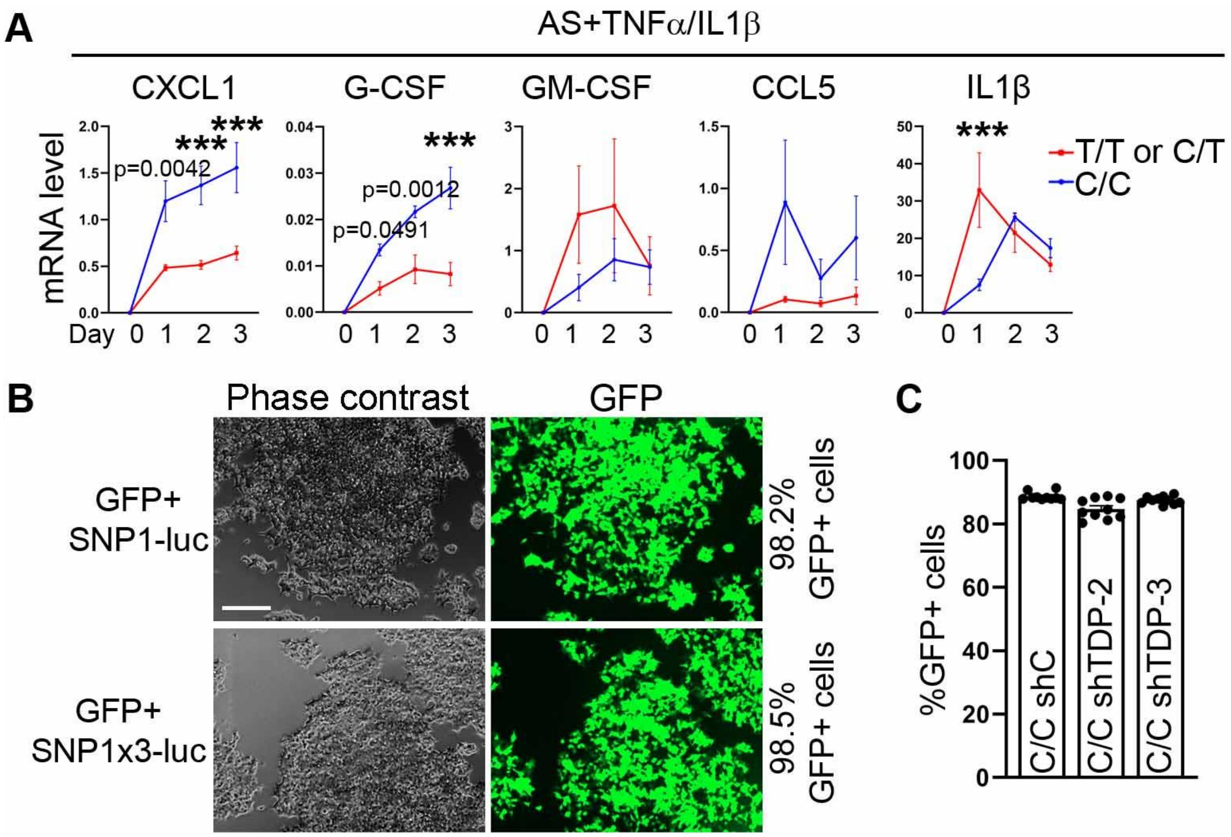

Fig. S2 The induction of chemokines and cytokines in astrocytes treated with TNFα/IL1β, related to Fig 2, 3.

(A) Induction of chemokines and cytokines (CXCL1, G-CSF, GM-CSF, CCL5, IL1β) at mRNA level in C/C, T/T or C/T astrocytes treated with TNFα/IL1β for 0, 1, 2, and 3 days as revealed by qRT-PCR.

(B) Control GFP transfection efficiency for TDP43 overexpression (OE) in HEK293T cells.

(C) Transduction efficiency for TDP43 knockdown (KD) in iPSC-derived C/C astrocytes.

Error bars are SEM of the mean. ***$p < 0.001$, analyzed by two-way ANOVA followed by Bonferroni's multiple comparisons test. 2 isogenic pairs were used, with 2 iPSC lines per group. n=8 independent experiments for (A), n=10 images for each condition for (C). Scale bar: 200 µm for (B).

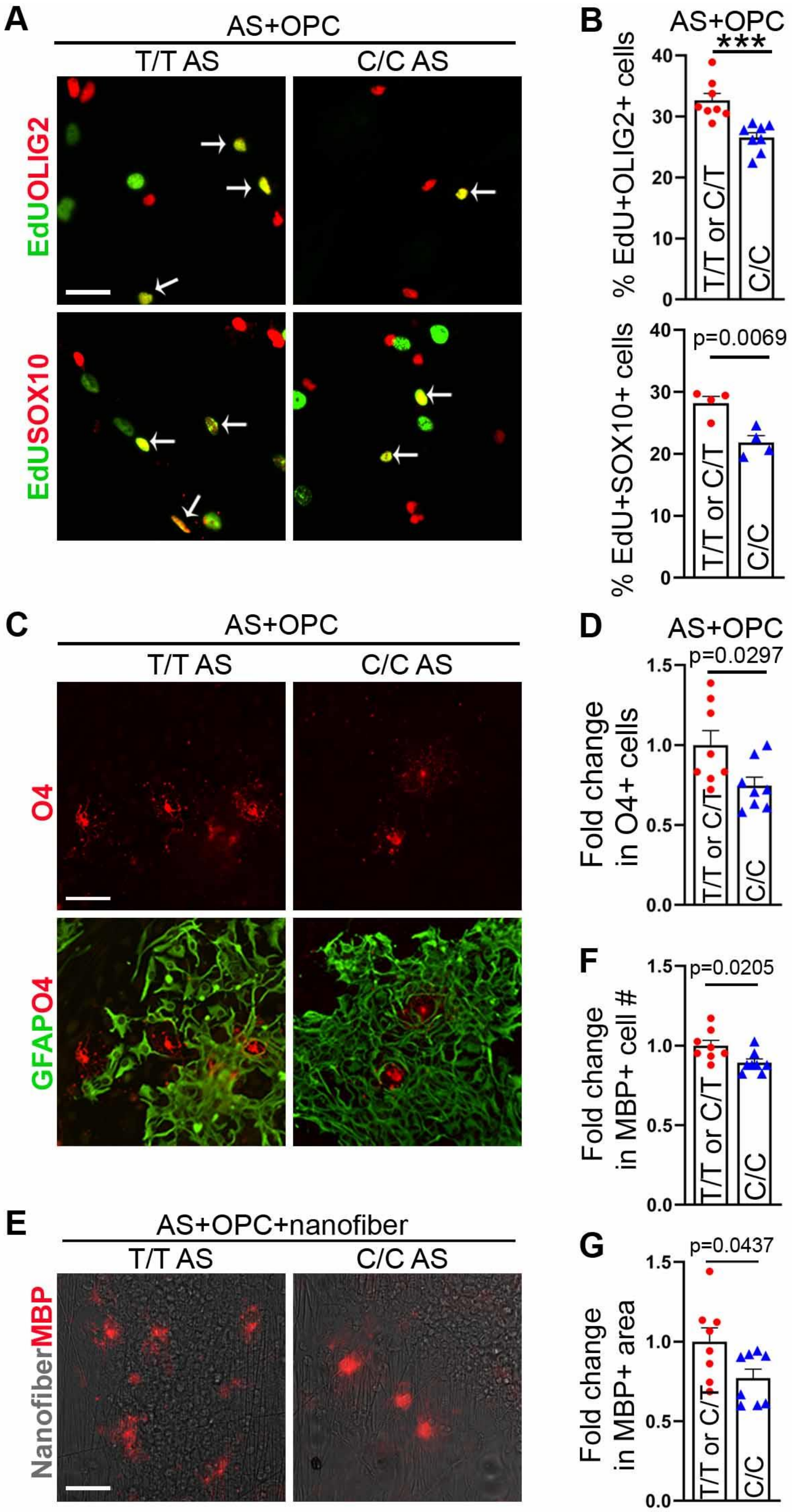

A
AS+OPC
T/T AS
C/C AS
EdUOLIG2
EdUSOX10
B
AS+OPC
% EdU+OLIG2+ cells
***
T/T or C/T
C/C
% EdU+SOX10+ cells
p=0.0069
C
AS+OPC
T/T AS
C/C AS
O4
GFAPO4
D
AS+OPC
p=0.0297
Fold change in O4+ cells
F
p=0.0205
Fold change in MBP+ cell #
E
AS+OPC+nanofiber
T/T AS
C/C AS
NanofiberMBP
G
p=0.0437
Fold change in MBP+ area

Fig. S3 C/C astrocytes induce mild inhibition of OPC proliferation and myelination in the absence of cytokine treatment, related to Fig 4.

(A, B) C/C astrocytes (AS) induce mild inhibition of OPC proliferation in the absence of cytokine treatment, compared to T/T or C/T astrocytes. C/C, T/T or C/T astrocytes were co-cultured with OPCs in the absence of cytokine treatment. The proliferation of OPC was evaluated by EdU labeling 1 day after co-culture. Arrows point to the EdU^+OLIG2^+ or EdU^+SOX10^+ proliferative OPCs. The percentage of EdU^+OLIG2^+ cells and EdU^+SOX10^+ cells in C/C, T/T or C/T AS is shown in panel B.
(C, D) Co-culture with C/C astrocytes leads to mildly reduced $O4^+$ cell number (#) in the absence of cytokine treatment. C/C, T/T or C/T astrocytes were co-cultured with OPCs in the absence of cytokine treatment. Mildly reduced population of OPCs in co-cultures with C/C astrocytes was revealed by decreased # of $O4^+$ cells 6 days after co-culture. Astrocytes in the co-culture were stained for GFAP. The fold change in $O4^+$ cell # is relative to that in co-cultures with T/T or C/T astrocytes in panel D.
(E-G) C/C astrocytes induce mild reduction of MBP^+ oligodendrocyte # in the absence of cytokine treatment, compared to T/T or C/T astrocytes. The # and area of MBP^+ oligodendrocytes were evaluated by staining for MBP in astrocyte-OPC-nanofiber co-cultures 20 days after co-culture (E). The fold change in MBP^+ cell # or MBP^+ area is relative to that in co-cultures with T/T or C/T astrocytes in (F, G).
Error bars are SEM of the mean. p values are indicated in the graphs or labelled as *** when $p < 0.001$, analyzed by two-tailed student’s t-test for (B, D, F, G). 2 isogenic pairs were used, with 2 iPSC lines per group. n=8 independent experiments for (B, D, F, G). Scale bar: 50 µm for (A, C, E).

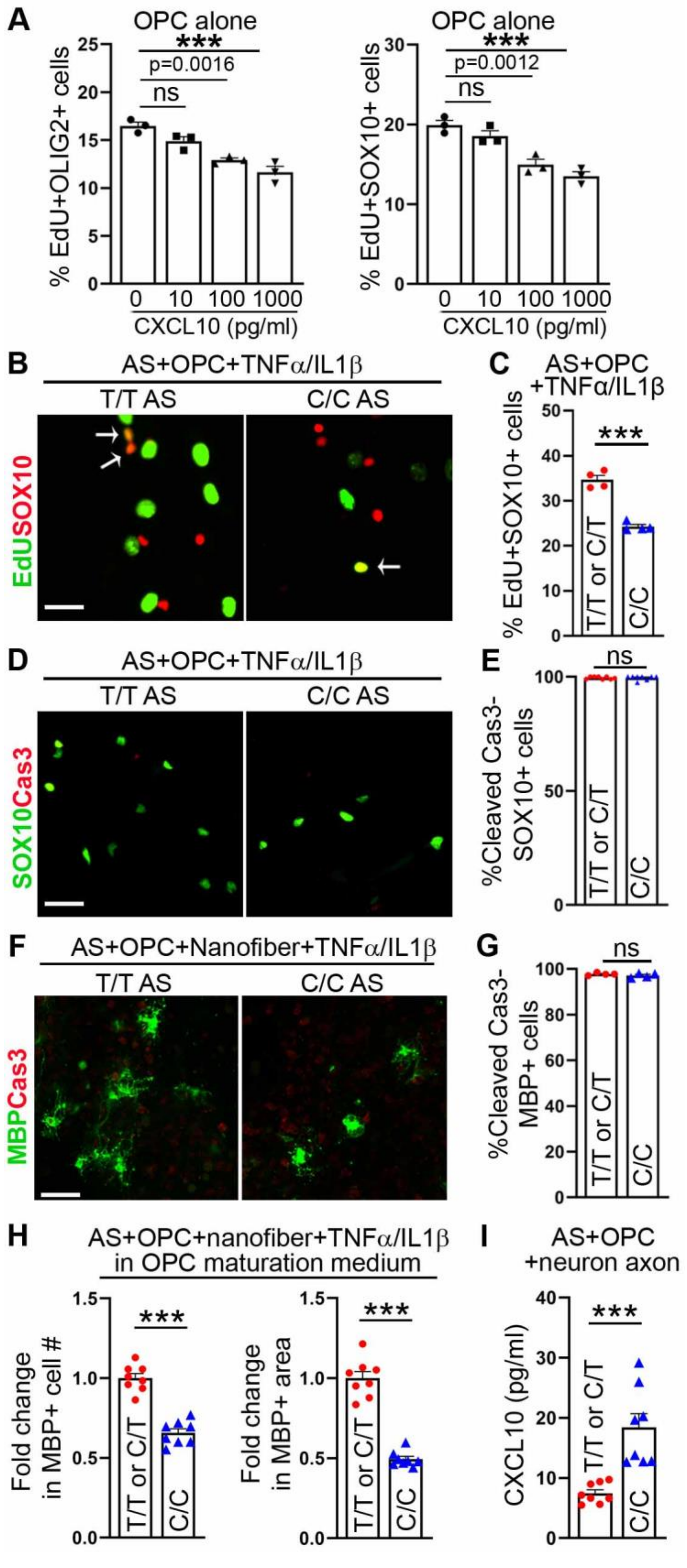
A
OPC alone
% EdU+OLIG2+ cells
***
p=0.0016
ns
0 10 100 1000
CXCL10 (pg/ml)
OPC alone
% EdU+SOX10+ cells
***
p=0.0012
ns
0 10 100 1000
CXCL10 (pg/ml)
B
AS+OPC+TNFα/IL1β
T/T AS
C/C AS
EdUSOX10
C
AS+OPC
+TNFα/IL1β
% EdU+SOX10+ cells
***
T/T or C/T
C/C
D
AS+OPC+TNFα/IL1β
T/T AS
C/C AS
SOX10Cas3
E
ns
%Cleaved Cas3-
SOX10+ cells
T/T or C/T
C/C
F
AS+OPC+Nanofiber+TNFα/IL1β
T/T AS
C/C AS
MBPCas3
G
ns
%Cleaved Cas3-
MBP+ cells
T/T or C/T
C/C
H
AS+OPC+nanofiber+TNFα/IL1β
in OPC maturation medium
***
Fold change
in MBP+ cell #
T/T or C/T
C/C
***
Fold change
in MBP+ area
T/T or C/T
C/C
I
AS+OPC
+neuron axon
***
CXCL10 (pg/ml)
T/T or C/T
C/C

Fig. S4 C/C astrocytes do not induce more OPC or oligodendrocyte cell death than T/T or C/T astrocytes in astrocyte-OPC co-cultures treated with cytokines, related to Fig 4.

(A) CXCL10 inhibits OPC proliferation. OPCs were treated with 0, 10, 100 or 1000 pg/ml CXCL10 for 1 day. OPC proliferation rate was indicated as the percentage of $EdU^{+}OLIG2^{+}$ cells (left) or $EdU^{+}SOX10^{+}$ cells (right).

(B, C) Cytokine-treated C/C astrocytes inhibit OPC proliferation, compared to T/T or C/T astrocytes. The proliferation of OPC was evaluated by EdU labeling 1 day after co-culture. Arrows point to the $EdU^{+}SOX10^{+}$ cells that represent proliferative OPCs (B). The percentage of $EdU^{+}SOX10^{+}$ cells in T/T or C/T and C/C astrocyte-OPC co-cultures treated with TNFα/IL1β is shown in panel C.

(D, E) C/C astrocytes do not include substantial OPC apoptosis in astrocyte-OPC co-cultures treated with TNFα/IL1β. Representative images of cleaved caspase-3 (Cas3) and SOX10 staining of TNFα/IL1β-treated co-cultures containing C/C, T/T or C/T astrocytes (C). The percentage of cleaved $Cas3^{-}SOX10^{+}$ cells in total $SOX10^{+}$ cells is shown in panel D.

(F, G) C/C astrocytes do not induce considerable oligodendrocyte apoptosis in TNFα/IL1β-treated astrocyte-OPC co-cultures on nanofibers. Representative images of cleaved caspase-3 (Cas3) and MBP staining of oligodendrocytes from TNFα/IL1β-treated co-cultures containing C/C, T/T or C/T astrocytes (E). The percentage of cleaved $Cas3^{-}MBP^{+}$ cells in total MBP^{+} cells is shown in panel F.

(H) C/C astrocytes reduce MBP^{+} cell # or MBP^{+} area in TNFα/IL1β-treated astrocyte-OPC-nanofiber co-cultures maintained in glial maturation medium, compared to T/T or C/T astrocytes. The astrocyte-OPC-nanofiber co-cultures were maintained in glial maturation medium for 20 days and treated with TNFα/IL1β. The fold change in MBP^{+} cell # or MBP^{+} area is relative to that in co-cultures with T/T or C/T astrocytes.

(I) ELISA detection of CXCL10 protein levels in C/C, T/T or C/T astrocytes co-cultured with OPC and neurons for 20 days.

Error bars are SEM of the mean. p values are indicated in the graphs or labelled as *** when $p < 0.001$, analyzed by one-way ANOVA followed by Tukey's multiple comparison test for (A), two-tailed student's t-test for (C, E, G-I). ns: not statistically significant ($p>0.05$). 2 isogenic pairs were used, with 2 iPSC lines per group for (C, E, G-I). n=3 independent experiments for (A), n=8 independent experiments for (E, H, I), n=4 independent experiments for (C, G). Scale bar: 50 µm for (B, D, F).

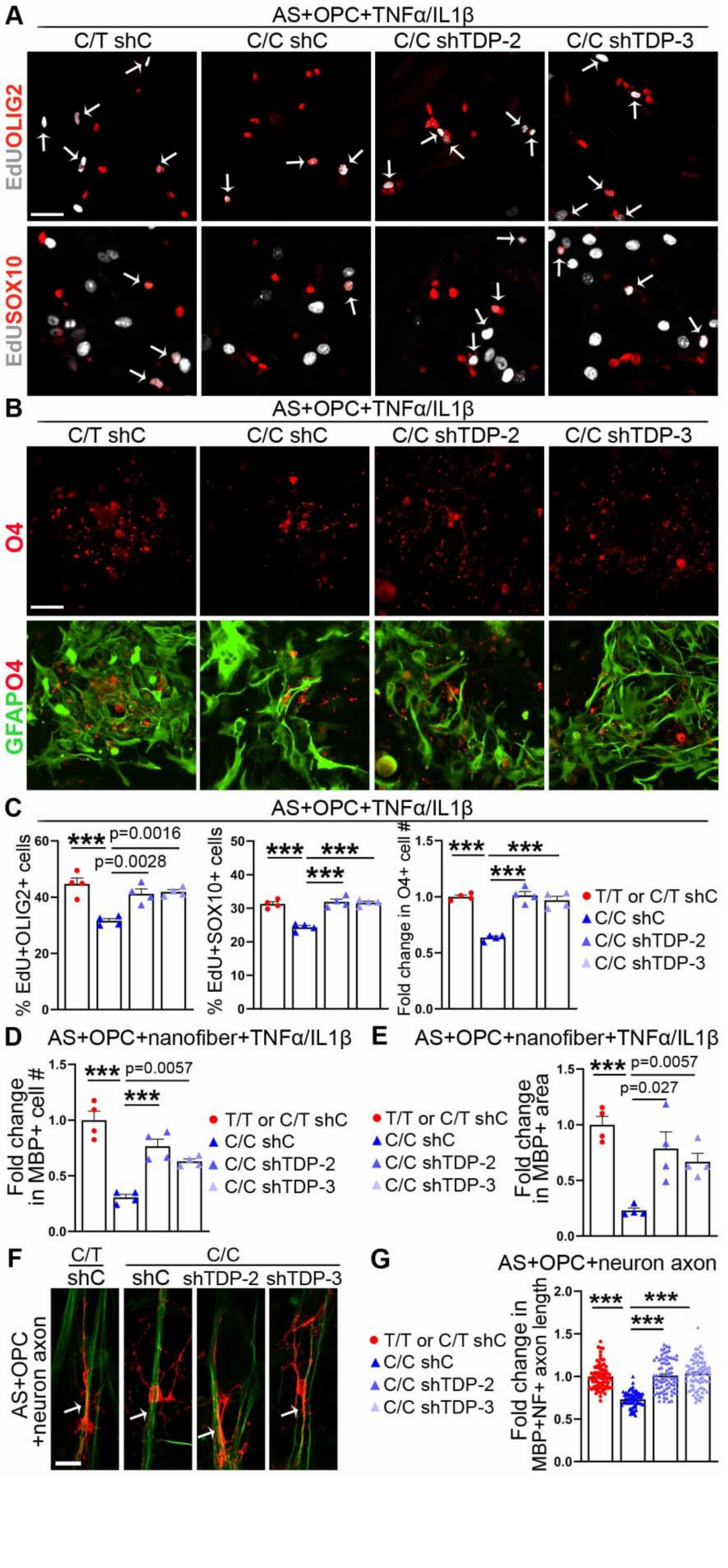
A
AS+OPC+TNFα/IL1β
C/T shC
C/C shC
C/C shTDP-2
C/C shTDP-3
EdUOLIG2
EdUSOX10
B
AS+OPC+TNFα/IL1β
C/T shC
C/C shC
C/C shTDP-2
C/C shTDP-3
O4
GFAPO4
C
AS+OPC+TNFα/IL1β
% EdU+OLIG2+ cells
p=0.0016
p=0.0028
% EdU+SOX10+ cells
Fold change in O4+ cell #
T/T or C/T shC
C/C shC
C/C shTDP-2
C/C shTDP-3
D
AS+OPC+nanofiber+TNFα/IL1β
Fold change in MBP+ cell #
p=0.0057
T/T or C/T shC
C/C shC
C/C shTDP-2
C/C shTDP-3
E
AS+OPC+nanofiber+TNFα/IL1β
Fold change in MBP+ area
p=0.0057
p=0.027
T/T or C/T shC
C/C shC
C/C shTDP-2
C/C shTDP-3
F
C/T
shC
C/C
shC
shTDP-2
shTDP-3
AS+OPC +neuron axon
G
AS+OPC+neuron axon
Fold change in MBP+NF+ axon length
T/T or C/T shC
C/C shC
C/C shTDP-2
C/C shTDP-3

Fig S5 Knockdown of TDP-43 in C/C astrocytes promotes OPC proliferation and myelination, related to Fig 4, 6.

(A-C) Knockdown (KD) of TDP-43 in C/C astrocytes treated with TNFα/IL1β promotes OPC proliferation and increases co-cultured OPC cell #. The proliferation of OPC was evaluated by EdU labeling 1 day after co-culture. Arrows point to the $EdU^{+}OLIG2^{+}$ or $EdU^{+}SOX10^{+}$ proliferative OPCs (A). The percentage of $EdU^{+}OLIG2^{+}$ cells (left) or $EdU^{+}SOX10^{+}$ cells (middle) in OPCs co-cultured with control (shC) T/T or C/T, control C/C, or TDP-43 KD (shTDP-2 or shTDP-3) C/C astrocytes is shown in panel C (left and middle). Increased $O4^{+}$ OPC cell # in co-cultures with TNFα/IL1β-treated TDP-43 KD C/C astrocytes, compared to that in co-cultures with TNFα/IL1β-treated control C/C astrocytes, was detected 6 days after co-culture. Astrocytes in the co-cultures were stained for GFAP (B). The fold change of $O4^{+}$ cell # is relative to the $O4^{+}$ cell # in co-cultures with control T/T or C/T astrocytes in panel C (right).

(D, E) KD of TDP-43 in C/C astrocytes treated with TNFα/IL1β increases the MBP^{+} cell # and MBP^{+} area in astrocyte-OPC-nanofiber co-cultures. The fold change in MBP^{+} cell # (D) or MBP^{+} area (E) is relative to that in co-cultures with TNFα/IL1β-treated control T/T or C/T astrocytes in panel E and F.

(F, G) KD of TDP-43 in C/C astrocytes increases $MBP^{+}NF^{+}$ axon length in astrocyte-neuron-OPC co-cultures. Astrocyte-neuron-OPC co-cultures were stained for MBP and NF to measure $MBP^{+}NF^{+}$ axon length 20 days after co-culture. Examples of the $MBP^{+}NF^{+}$ axons are pointed by arrows (F). The fold change in $MBP^{+}NF^{+}$ axon length is relative to that in co-cultures with control T/T or C/T astrocytes in panel G.

Error bars are SEM of the mean. p values are indicated in the graphs or labelled as *** when $p < 0.001$, analyzed by one-way ANOVA followed by Tukey's multiple comparison test. 2 isogenic pairs were used, with 2 iPSC lines per group. n=4 independent experiments for (C-E), n=80 MBP^{+} cells from 4 independent experiments for (G). Scale bar: 50 μm for (A, B), 20 μm for (F).

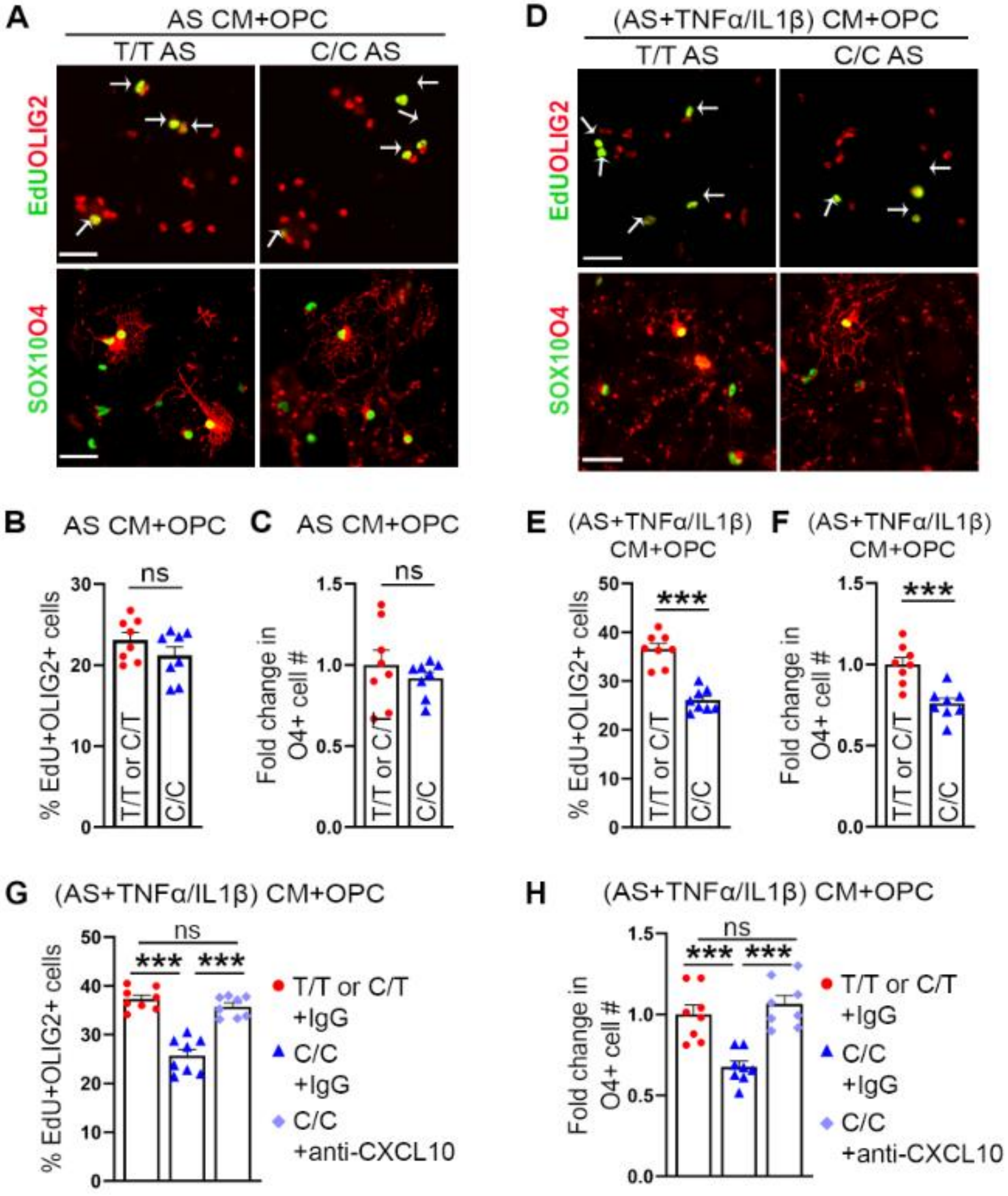
A
AS CM+OPC
T/T AS
C/C AS
EdUOLIG2
SOX10O4
D
(AS+TNFα/IL1β) CM+OPC
T/T AS
C/C AS
EdUOLIG2
SOX10O4
B
AS CM+OPC
% EdU+OLIG2+ cells
ns
T/T or C/T
C/C
C
AS CM+OPC
Fold change in O4+ cell #
ns
T/T or C/T
C/C
E
(AS+TNFα/IL1β) CM+OPC
% EdU+OLIG2+ cells
***
T/T or C/T
C/C
F
(AS+TNFα/IL1β) CM+OPC
Fold change in O4+ cell #
***
T/T or C/T
C/C
G
(AS+TNFα/IL1β) CM+OPC
% EdU+OLIG2+ cells
ns
***
***
T/T or C/T +IgG
C/C +IgG
C/C +anti-CXCL10
H
(AS+TNFα/IL1β) CM+OPC
Fold change in O4+ cell #
ns
***
***
T/T or C/T +IgG
C/C +IgG
C/C +anti-CXCL10

Fig. S6 Conditioned medium from cytokine-treated C/C astrocytes inhibits OPC proliferation, related to Fig 5, 6.

(A-C) Conditioned medium (CM) collected from C/C astrocytes without cytokine treatment has no effect on OPC proliferation and cell number, compared to CM from T/T or C/T astrocytes. CM collected from C/C, T/T or C/T astrocytes without cytokine treatment was used to treat OPCs. OPC proliferation was evaluated by EdU labeling 1 day after CM treatment. Arrows point to the $EdU^{+}OLIG2^{+}$ proliferative OPCs (panel A, upper). The percentage of $EdU^{+}OLIG2^{+}$ cells in OPCs treated with CM from C/C, T/T or C/T astrocytes is shown in panel B. The fold change in $O4^{+}$ cell #, relative to that in OPCs treated with CM from T/T or C/T astrocytes, is shown in panel C.

(D-F) CM collected from cytokine-treated C/C astrocytes inhibit OPC proliferation and cell number. The proliferation of OPC was evaluated by EdU labeling 1 day after CM treatment. Arrows point to the $EdU^{+}OLIG2^{+}$ cells that represent proliferative OPCs (panel D, upper). The percentage of $EdU^{+}OLIG2^{+}$ cells in OPCs treated with CM from TNFα/IL1β-treated C/C, T/T or C/T astrocytes is shown in panel E. The fold change in $O4^{+}$ cell #, relative to that in OPCs treated with CM from TNFα/IL1β-treated T/T or C/T astrocytes, is shown in panel F.

(G) The percentage of $EdU^{+}OLIG2^{+}$ cells in OPCs treated with CM collected from C/C astrocytes treated with cytokine together with IgG or the anti-CXCL10 neutralizing antibody. CM from T/T or C/T astrocytes treated with cytokine together with IgG was included as a control.

(H) The fold change in $O4^{+}$ cell # in OPCs treated with CM collected from C/C astrocytes treated with cytokine together with IgG or the anti-CXCL10 neutralizing antibody. The fold change in $O4^{+}$ cell # is relative to that in OPCs treated with CM from T/T or C/T astrocytes treated with cytokine together with IgG.

Error bars are SEM of the mean. ***$p < 0.001$ by two-tailed student's t-test for (B, C, E, F) and one-way ANOVA followed by Tukey's multiple comparison test for (G, H). ns: not statistically significant ($p>0.05$). 2 isogenic pairs were used, with 2 iPSC lines per group. n=8 independent experiments for (B, C, E-H). Scale bar: 50 µm for (A, D).

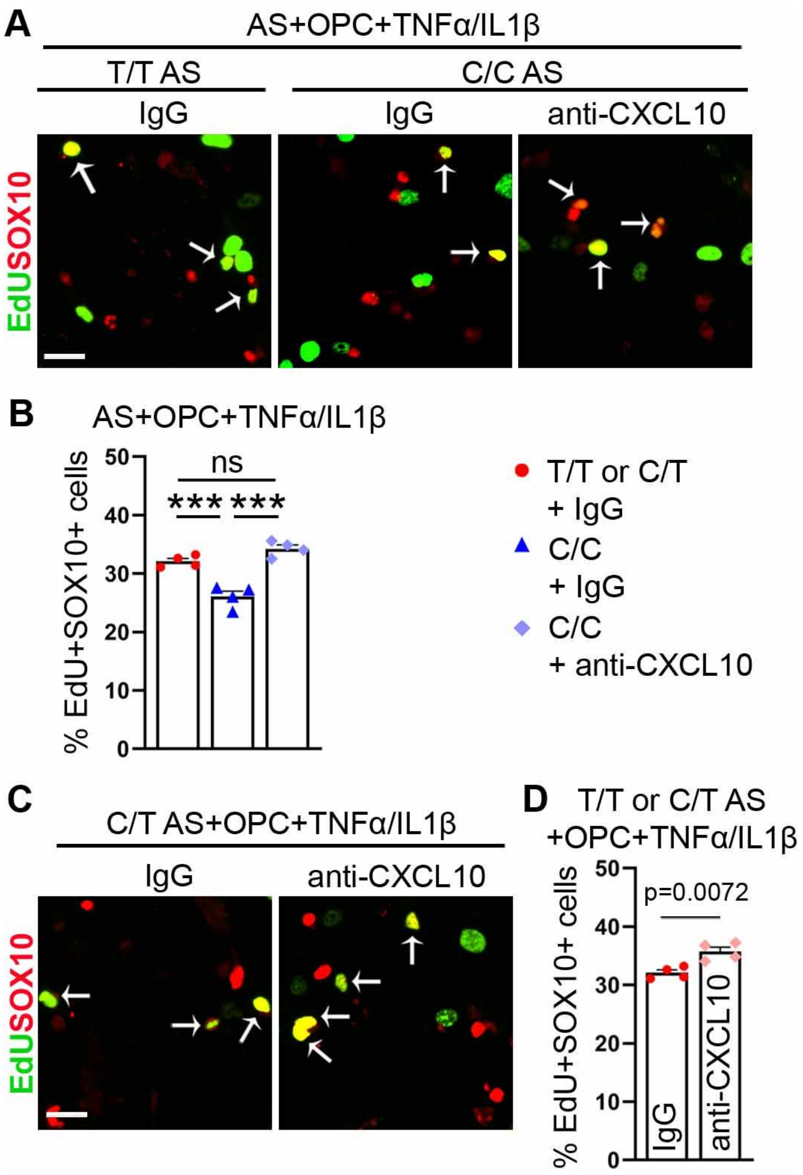
A
AS+OPC+TNFα/IL1β
T/T AS
C/C AS
IgG
IgG
anti-CXCL10
EdUSOX10
B
AS+OPC+TNFα/IL1β
% EdU+SOX10+ cells
50
40
30
20
10
0
ns
*** ***
T/T or C/T
+ IgG
C/C
+ IgG
C/C
+ anti-CXCL10
C
C/T AS+OPC+TNFα/IL1β
IgG
anti-CXCL10
EdUSOX10
D
T/T or C/T AS
+OPC+TNFα/IL1β
% EdU+SOX10+ cells
50
40
30
20
10
0
p=0.0072
IgG
anti-CXCL10

Fig. S7 A CXCL10 neutralizing antibody increases OPC proliferation in astrocyte-OPC co-cultures, related to Fig 6.

(A) Treatment with the CXCL10 neutralizing antibody rescued OPC proliferation in astrocyte-OPC co-cultures with C/C astrocytes treated with TNFα/IL1β. Representative images of EdU and SOX10 double staining after 1-day co-culture in C/C astrocyte-OPC co-cultures treated with IgG plus TNFα/IL1β or anti-CXCL10 antibody plus TNFα/IL1β. T/T or C/T astrocyte-OPC co-cultures treated with IgG plus TNFα/IL1β were included as a control.

(B) The percentage of $EdU^{+}SOX10^{+}$ cells in C/C astrocyte-OPC co-cultures treated with IgG plus TNFα/IL1β or anti-CXCL10 plus TNFα/IL1β. T/T or C/T astrocyte-OPC co-cultures treated with IgG plus TNFα/IL1β were included as a control.

© Treatment with the CXCL10 neutralizing antibody increased OPC proliferation in astrocyte-OPC co-cultures with T/T or C/T astrocytes treated with TNFα/IL1β. Representative images of EdU and SOX10 double staining after 1-day co-culture in T/T or C/T astrocyte-OPC co-cultures treated with IgG plus TNFα/IL1β or anti-CXCL10 antibody plus TNFα/IL1β.

(D) The percentage of $EdU^{+}SOX10^{+}$ cells in T/T or C/T astrocyte-OPC co-cultures treated with IgG plus TNFα/IL1β or anti-CXCL10 plus TNFα/IL1β.

Error bars are SEM of the mean. p values are indicated in the graphs or labelled as *** when $p < 0.001$, analyzed by one-way ANOVA followed by Tu’ey's multiple comparison test for (B), two-tailed student’s t-test for (D). ns: not statistically significant ($p>0.05$). 2 isogenic pairs were used, with 2 iPSC lines per group. n=4 independent experiments.

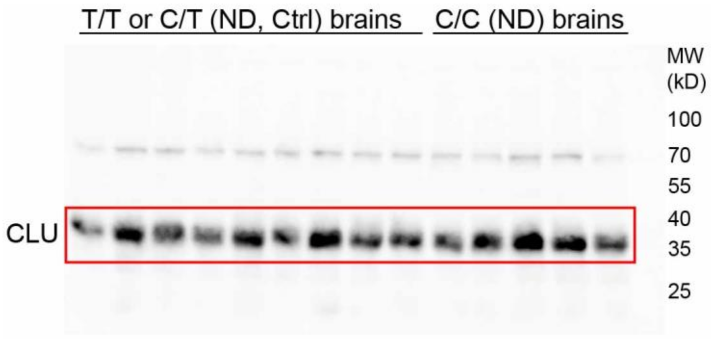

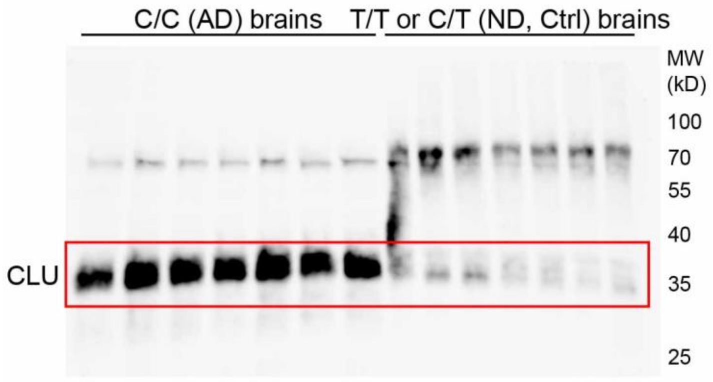

Fig. S8 Elevated CLU protein level in C/C vs T/T or C/T brains, related to Fig 7. Uncropped images for Western blot of CLU in T/T or C/T ND brain, C/C ND brain, and C/C AD brain tissues. The T/T or C/T ND brain tissues were included as controls for C/C ND brain tissues in blot 1 and for C/C AD brain tissues in blot 2.

Supplementary Tables

Supplementary Table 1 List of human iPSCs and astrocytes used in the study, related to Fig 1, 2.

iPSC lines	Astrocyte lines	ND, AD, or CRISPR	Gender	Age	ApoE genotype	Karyotype	Source	CLU rs11136000
iPSC1-C/T	AS1-C/T	AD	Female	70	E3/3	Normal	UCI	C/T
iPSC1-C/C	AS1-C/C	CRISPR	Female	70	E3/3	Normal	This paper	C/C
iPSC2-C/C	AS2-C/C	AD	Female	60	E3/3	Normal	Wang et al.	C/C
iPSC2-T/T	AS2-T/T	CRISPR	Female	60	E3/3	Normal	This paper	T/T
iPSC3-C/C	AS3-C/C	ND	Male	71	E3/3	Normal	Wang et al.	C/C
iPSC3-C/T	AS3-C/T	CRISPR	Male	71	E3/3	Normal	This paper	C/T
iPSC3-T/T	AS3-T/T	CRISPR	Male	71	E3/3	Normal*	This paper	T/T
**iPSC lines**	**Astrocyte lines**	**ND, AD, or CRISPR**	**Gender**	**Age**	**ApoE genotype**	**Karyotype**	**Source**	**CLU SNPs**
iPSC3-C/C (WT)	AS3-C/C (WT)	ND	Male	71	E3/3	Normal	Wang et al.	WT
SNP1 KO-C1	SNP1 KO-C1	CRISPR	Male	71	E3/3	Normal	This paper	SNP1 KO
SNP1 KO-C2	SNP1 KO-C2	CRISPR	Male	71	E3/3	Normal	This paper	SNP1 KO
SNP2 KO-C1	SNP2 KO-C1	CRISPR	Male	71	E3/3	Normal	This paper	SNP2 KO
SNP2 KO-C2	SNP2 KO-C2	CRISPR	Male	71	E3/3	Normal	This paper	SNP2 KO
SNP3 KO-C1	SNP3 KO-C1	CRISPR	Male	71	E3/3	Normal	This paper	SNP3 KO
SNP3 KO-C2	SNP3 KO-C2	CRISPR	Male	71	E3/3	Normal	This paper	SNP3 KO
SNP4 KO-C1	SNP4 KO-C1	CRISPR	Male	71	E3/3	Normal	This paper	SNP4 KO
SNP4 KO-C2	SNP4 KO-C2	CRISPR	Male	71	E3/3	Normal	This paper	SNP4 KO

SNP5 KO-C1	SNP5 KO-C1	CRISPR	Male	71	E3/3	Normal	This paper	SNP5 KO
SNP5 KO-C2	SNP5 KO-C2	CRISPR	Male	71	E3/3	Normal	This paper	SNP5 KO

* Normal male karyotype with minor clone missing Y chromosome.

Supplementary Table 2 The karyotype of SNP KO iPSCs, related to Table S1.

iPSC lines	SNP1 KO-C1	SNP1 KO-C1	SNP1 KO-C2	SNP1 KO-C2	SNP2 KO-C1	SNP2 KO-C1	SNP2 KO-C2	SNP2 KO-C2	SNP3 KO-C1	SNP3 KO-C1
Genes	Copy Number	p-Value	Copy Number	p-Value	Copy Number	p-Value	Copy Number	p-Value	Copy Number	p-Value
chr1q	1.93	0.977	1.76	0.994	1.95	0.994	2.01	1.000	2.05	0.997
chr4p	2.04	0.583	1.93	0.350	2.03	0.961	2.05	0.991	2.03	0.999
chr8q	1.91	0.993	1.91	0.977	1.9	0.997	2.06	0.998	1.86	0.824
chr10p	1.81	0.998	1.69	0.881	1.78	0.989	2.12	0.364	1.98	0.928
chr12p	1.78	0.997	1.7	0.995	1.78	0.956	2.18	0.318	1.92	0.996
chr17q	1.84	0.998	1.71	0.980	2.25	0.530	1.97	0.998	1.98	0.995
chr18q	1.74	0.936	2.12	0.491	2.02	0.975	1.96	0.979	2.12	0.992
chr20q	1.81	0.999	1.9	0.925	1.98	0.995	1.95	0.999	2.2	0.913
chrXp	1.75	0.968	1.8	0.997	2.03	0.996	2.01	0.999	2.03	0.998
**iPSC lines**	**SNP3 KO-C2**	**SNP3 KO-C2**	**SNP4 KO-C1**	**SNP4 KO-C1**	**SNP4 KO-C2**	**SNP4 KO-C2**	**SNP5 KO-C1**	**SNP5 KO-C1**	**SNP5 KO-C2**	**SNP5 KO-C2**
Genes	Copy Number	p-Value	Copy Number	p-Value	Copy Number	p-Value	Copy Number	p-Value	Copy Number	p-Value
chr1q	2.13	0.943	2.08	0.754	1.96	0.984	2.07	0.907	1.98	1.000
chr4p	2.15	0.990	2.03	0.692	2.01	0.637	2.06	0.889	2.1	0.992
chr8q	1.94	0.747	1.9	0.914	1.97	0.977	1.98	0.976	2.03	0.998
chr10p	2.09	0.998	1.86	0.821	1.76	0.938	2.05	0.908	2.04	0.961
chr12p	1.99	0.879	1.76	0.133	1.87	0.998	2	0.929	1.94	0.995
chr17q	1.88	0.087	1.89	0.915	2.14	0.041	2.12	0.721	2.18	0.970
chr18q	2.03	0.995	1.95	0.857	1.86	0.984	1.95	0.896	1.9	0.998
chr20q	2.12	0.997	1.94	0.932	1.9	0.998	1.93	0.798	1.97	0.999
chrXp	2.01	0.998	2.02	0.917	1.86	0.998	1.99	0.994	2.05	0.623

Supplementary Table 4 List of nuclear proteins exhibited different binding between C and T alleles detected by mass spectrometry, related to Fig 3.

Protein	Sequence coverage				Intensity				p-value
	C1	C2	T1	T2	C1	C2	T1	T2	
TDP43	16.4	16.4	0	0	69817000	65503000	0	0	0.001015
GAL7	19.9	19.9	0	0	61764000	61395000	0	0	8.98E-06
PCBP2	12.7	22.8	12.7	12.7	9715200	35263000	0	0	0.22038

DDX3X	4.6	6.5	1.6	1.6	15548000	54380000	0	0	0.213536
HP1BP3	2	4	2	2	0	14895000	0	0	0.42265
TAF15	4.6	6.9	2.4	0	2376400	2537700	0	0	0.001076
DESMIN	10.2	7.2	7.2	10	0	0	0	435170000	0.42265
GSN	30.8	30.8	25.8	25.8	0	0	16035000	16118000	6.66E-06

Supplementary Table 5 List of human brain tissues used in the study, related to Fig 7.

**Human brain tissues**	**CLU rs1113600**	**ApoE genotype**	**Gender**	**Age**	**AD status**
S1	T/T	E3/3	Male	61	No
S2	T/T	E3/3	Male	76	No
S3	T/T	E3/3	Male	80	No
S4	T/T	E3/3	Male	91	No
S5	C/T	E3/3	Female	59	No
S6	C/T	E3/3	Male	82	No
S7	C/T	E3/3	Female	82	No
S8	C/T	E3/3	Female	90	No
S9	C/T	E3/3	Female	95	No
S10	C/C	E3/3	Female	75	No
S11	C/C	E3/3	Female	76	No
S12	C/C	E3/3	Male	89	No
S13	C/C	E3/3	Male	63	No
S14	C/C	E3/3	Male	79	No
S15	C/C	E3/3	Male	86	No
S16	C/C	E3/3	Male	90	No
S17	C/C	E3/3	Female	87	Yes
S18	C/C	E3/3	Male	87	Yes
S19	C/C	E3/3	Female	86	Yes
S20	C/C	E3/3	Female	87	Yes
S21	C/C	E3/3	Female	≥90	Yes
S22	C/C	E3/3	Male	85	Yes
S23	C/C	E3/3	Female	≥90	Yes
S24	C/C	E3/3	Male	≥90	Yes
S25	C/C	E3/3	Female	86	Yes

Supplementary Table 6 Oligonucleotide sequences for gene editing and PCR, related to STAR Methods.

**Oligonucleotide Name**	**Oligonucleotide Sequence**	**CLU isoforms**

CLU SNP1 KO sgRNA 1	5’-UCUCUUUUAAGCAUCUGGGU-3’	N/A
CLU SNP1 KO sgRNA 2	5’-UGAGAGGGUAGUCAUUGAGU-3’	N/A
CLU SNP2 KO sgRNA 1	5’-ACAGAACCAACAGGACGAUG-3’	217
CLU SNP2 KO sgRNA 2	5’-UCUCCCACUAGGGAUGCAGA-3’	202, 215
CLU SNP3 KO sgRNA 1	5’-UUCUACCCUACUCUGACCAA-3’	211
CLU SNP3 KO sgRNA 2	5’-AGGGAAGAGCUGAAACACUG-3’	211
CLU SNP4 KO sgRNA 1	5’-CCUUGACCAUCCCACUGGAG-3’	203
CLU SNP4 KO sgRNA 2	5’-AUCACAUGCUAGUUCCAAGC-3’	203
CLU SNP5 KO sgRNA 1	5’-CCUCUGACCUCAUCACCCUG-3’	N/A
CLU SNP5 KO sgRNA 2	5’-UAAAAACCCAGAUCAGACAU-3’	N/A
rs11136000 C to T sgRNA	5’-CGUUAGAGAGUUUUGAUAGC-3’	N/A
rs11136000 T to C sgRNA	5’-CGUUAGAGAAUUUUGAUAGC-3’	N/A
rs11136000 C to T ssODN	5’-TCCTGGCGTGCAAAGGGAATGGCAG GCATTCAGCACCAAAGCCACACCAGCT ATCAAAATTCTCTAACGGGCCCTTGCCA CTTGACCCAATAATTCTGTAAGAATCTG TCTCAGGCCAGG-3’	N/A
rs11136000 T to C ssODN	5’-TCCTGGCGTGCAAAGGGAATGGCAG GCATTCAGCACCAAAGCCACACCAGCT ATCAAAACTCTCTAACGGGCCCTTGCC ACTTGACCCAATAATTCTGTAAGAATCT GTCTCAGGCCAGG-3’	N/A
CLU-SNP1-Forward	5’-TGTCAGGGGATTCCTTTGAGATA-3’	N/A
CLU-SNP1-Reverse	5’-ATGCAGGTCTGTTTCAGGCA-3’	N/A
CLU-SNP2-Forward	5’-CTGGCTTTGTCTCTCTGGCAT-3’	N/A
CLU-SNP2-Reverse	5’-TCCTCTGCAATGTGCACCTA-3’	N/A
CLU-SNP3-Forward	5’-ATTGCCTGAGCCCTGAAGT-3’	N/A
CLU-SNP3-Reverse	5’-GTGCTTTTTGCGGTATTCCTGCAG-3’	N/A

CLU-SNP4-Forward	5’-CCTTGACAGCCCCTGAACTG-3’	N/A
CLU-SNP4-Reverse	5’-GAAGTAGGGCGACCGTGAGA-3’	N/A
CLU-SNP5-Forward	5’-CCAGAATTGGAGGCATGATG-3’	N/A
CLU-SNP5-Reverse	5’-GGAGGAGTATGTTCTGGAGGTC-3’	N/A

*N/A means that the oligonucleotide is not located on any isoforms.

Supplementary Table 7: Oligonucleotide sequences for qPCR, related to STAR Methods.

Primer Name	Primer Sequence	CLU isoform coverage
hCLU-1-Forward	5’-GAGCGCAAGACACTGCTCA-3’	201, 202, 205, 206, 207, 211, 212, 215, 216, 217
hCLU-1-Reverse	5’-TTCCCTGGTCTCATTTAGGGC-3’	201, 202, 205, 206, 207, 211, 212, 215, 216, 217
hCLU-2-Forward	5’-TGTCTTGCGCTCTTCGTTTG-3’	201, 202, 203, 204, 205, 206, 207, 211, 212, 214, 215, 216, 217
hCLU-2-Reverse	5’-ACCAGACGGTCTCAGACAATG-3’	201, 202, 203, 204, 205, 206, 207, 211, 212, 214, 215, 216, 217
hCLU-3-Forward	5’- TCTTTCCCAAGTCCCGCATC-3’	201, 202, 209, 211, 213, 215, 217
hCLU-3-Reverse	5’- CACACAGTCCGGTCATCGTC -3’	201, 202, 209, 211, 213, 215, 217
hCLU-4-Forward	5’-TCAGTGACACCGGAAGGAAC-3’	201, 202, 208, 209, 215
hCLU-4-Reverse	5’-AACAACCCCTCCCAGGCTAA-3’	201, 202, 208, 209, 215
hGAPDH-Forward	5’-CCTGTTCGACAGTCAGCCG-3’	N/A
hGAPDH-Reverse	5’-CGACCAAATCCGTTGACTCC-3’	N/A
hMBP-Forward	5’-AAGGCCAGAGACCAGGATTT-3’	N/A
hMBP-Reverse	5’-TCCCTTGAATCCCTTGTGAG-3’	N/A
hCCDC25-Forward	5’-AGACCAAAGTCGAGCGGTTC-3’	N/A
hCCDC25-Reverse	5’-ATTGCCATCCTGATTTGAAGACA-3’	N/A

hCHRNA2-Forward	5’-ACCACCAACGTCTGGCTAAA-3’	N/A
hCHRNA2-Reverse	5’-CTCCCCATCTGCATTGTTGT-3’	N/A
hEPHX2-Forward	5’-ACTCCCTTCATACCAGCAAATC-3’	N/A
hEPHX2-Reverse	5’-GACTCAGGTTCTGTTCCAGTTC-3’	N/A
hESCO2-Forward	5’-TCTCCTAAGTCCACTGTCTATCC-3’	N/A
hESCO2-Reverse	5’-GAAGTTGACAGATCCCACAGAA-3’	N/A
hPBK-Forward	5’-TTGAAAGCCAGGAGGGTTCG-3’	N/A
hPBK-Reverse	5’-CTCAGTCCAGAGTCTCACCGC-3’	N/A
hPTK2B-Forward	5’-GGGAGGTCTATGAAGGTGTCTA-3’	N/A
hPTK2B-Reverse	5’-CTTCTCCTTGTTGTCCAGAGTG-3’	N/A
hSCARA3-Forward	5’-CATCTCCTTGACCCAGTCTATTT-3’	N/A
hSCARA3-Reverse	5’-TGGCAGAAAGAGCAGTTGT-3’	N/A
hSCARA5-Forward	5’-CGATTCGGGCAAGGCACT-3’	N/A
hSCARA5-Reverse	5’-CGGCATGTCCACAGTTTGTC-3’	N/A
hTRIM35-Forward	5’-CTCCCGTGTCTTCTCACAGG-3’	N/A
hTRIM35-Reverse	5’-CGAGCGTGTGTCGTGGTAG-3’	N/A
hCXCL10-Forward	5’-CCTGCAAGCCAATTTTGTCCA-3’	N/A
hCXCL10-Reverse	5’-TGATGGCCTTCGATTCTGGAT-3’	N/A
hCXCL1-Forward	5’-CTCAATCCTGCATCCCCCATA-3’	N/A
hCXCL1-Reverse	5’-TGTTCCTATAAGGGCAGGGC-3’	N/A
hG-CSF-Forward	5’-TCCAGGAGAAGCTGGTGAGTGA-3’	N/A
hG-CSF-Reverse	5’-CGCTATGGAGTTGGCTCAAGCA-3’	N/A
hGM-CSF-Forward	5’-GCGTCTCCTGAACCTGAGTAG-3’	N/A
hGM-CSF-Reverse	5’-TCGGCTCCTGGAGGTCAAA-3’	N/A

hCCL5-Forward	5’-GGCAGCCCTCGCTGTCATCC-3’	N/A
hCCL5-Reverse	5’-GCAGCAGGGTGTGGTGTCCG-3’	N/A
hIL1β-Forward	5’-GCAGGCCGCGTCAGTTGTTG-3’	N/A
hIL1β -Reverse	5’-CCCGGAGCGTGCAGTTCAGT-3’	N/A
